# On the origin of the translation system and the genetic code in the RNA world by means of natural selection, exaptation, and subfunctionalization

**DOI:** 10.1186/1745-6150-2-14

**Published:** 2007-05-31

**Authors:** Yuri I Wolf, Eugene V Koonin

**Affiliations:** 1 National Center for Biotechnology Information, National Library of Medicine, National Institutes of Health, Bethesda, MD 20894, USA

## Abstract

**Background:**

The origin of the translation system is, arguably, the central and the hardest problem in the study of the origin of life, and one of the hardest in all evolutionary biology. The problem has a clear catch-22 aspect: high translation fidelity hardly can be achieved without a complex, highly evolved set of RNAs and proteins but an elaborate protein machinery could not evolve without an accurate translation system. The origin of the genetic code and whether it evolved on the basis of a stereochemical correspondence between amino acids and their cognate codons (or anticodons), through selectional optimization of the code vocabulary, as a "frozen accident" or via a combination of all these routes is another wide open problem despite extensive theoretical and experimental studies. Here we combine the results of comparative genomics of translation system components, data on interaction of amino acids with their cognate codons and anticodons, and data on catalytic activities of ribozymes to develop conceptual models for the origins of the translation system and the genetic code.

**Results:**

Our main guide in constructing the models is the Darwinian Continuity Principle whereby a scenario for the evolution of a complex system must consist of plausible elementary steps, each conferring a distinct advantage on the evolving ensemble of genetic elements. Evolution of the translation system is envisaged to occur in a compartmentalized ensemble of replicating, co-selected RNA segments, i.e., in a RNA World containing ribozymes with versatile activities. Since evolution has no foresight, the translation system could not evolve in the RNA World as the result of selection for protein synthesis and must have been a by-product of evolution drive by selection for another function, i.e., the translation system evolved via the exaptation route. It is proposed that the evolutionary process that eventually led to the emergence of translation started with the selection for ribozymes binding abiogenic amino acids that stimulated ribozyme-catalyzed reactions. The proposed scenario for the evolution of translation consists of the following steps: binding of amino acids to a ribozyme resulting in an enhancement of its catalytic activity; evolution of the amino-acid-stimulated ribozyme into a peptide ligase (predecessor of the large ribosomal subunit) yielding, initially, a unique peptide activating the original ribozyme and, possibly, other ribozymes in the ensemble; evolution of self-charging proto-tRNAs that were selected, initially, for accumulation of amino acids, and subsequently, for delivery of amino acids to the peptide ligase; joining of the peptide ligase with a distinct RNA molecule (predecessor of the small ribosomal subunit) carrying a built-in template for more efficient, complementary binding of charged proto-tRNAs; evolution of the ability of the peptide ligase to assemble peptides using exogenous RNAs as template for complementary binding of charged proteo-tRNAs, yielding peptides with the potential to activate different ribozymes; evolution of the translocation function of the protoribosome leading to the production of increasingly longer peptides (the first proteins), i.e., the origin of translation. The specifics of the recognition of amino acids by proto-tRNAs and the origin of the genetic code depend on whether or not there is a physical affinity between amino acids and their cognate codons or anticodons, a problem that remains unresolved.

**Conclusion:**

We describe a stepwise model for the origin of the translation system in the ancient RNA world such that each step confers a distinct advantage onto an ensemble of co-evolving genetic elements. Under this scenario, the primary cause for the emergence of translation was the ability of amino acids and peptides to stimulate reactions catalyzed by ribozymes. Thus, the translation system might have evolved as the result of selection for ribozymes capable of, initially, efficient amino acid binding, and subsequently, synthesis of increasingly versatile peptides. Several aspects of this scenario are amenable to experimental testing.

**Reviewers:**

This article was reviewed by Rob Knight, Doron Lancet, Alexander Mankin (nominated by Arcady Mushegian), and Arcady Mushegian.

## Open peer review

This article was reviewed by Rob Knight, Doron Lancet, Alexander Mankin (nominated by Arcady Mushegian), and Arcady Mushegian.

*...there is no logical impossibility in the acquirement of any conceivable degree of perfection through natural selection*.

**Ch. Darwin **[1]

*...the origin of protein synthesis is a notoriously difficult problem*.

**F.H.C. Crick et al**. [2]

## Background

### The Darwin-Eigen cycle, the emergence of biological complexity, and the continuity principle

As first outlined by Darwin [1], the evolution of life is based on the triad of heredity (the property of progeny to resemble their parent(s)), variation (generation of variants as a result of errors during reproduction), and selection (differential reproduction of variants). The theory of self-replicating systems that was developed, primarily, by Eigen and coworkers in the 1970ies [3] revealed an important limit (hereinafter the Eigen threshold) on the relationships between the reproduction fidelity and the amount of information contained in the system. Simply put, if the product of the error (mutation) rate and the information capacity (genome size) is safely below one (i.e., less then one error per genome is expected to occur per replication cycle), most of the progeny will be exact copies of the parent, and reproduction of the system will be sustainable. If, in contrast, this value is significantly greater than one, most of the progeny will differ from the parent, and the system will not possess sufficiently faithful heredity to reproduce itself; in other words, a system whose fidelity drops below the Eigen threshold is headed for collapse resulting from an error catastrophe (a term and idea traceable to the early hypothesis of Orgel on the possible contribution of translation errors to aging [4]). It appears that the product of the replication fidelity and the genome size of modern life forms, from RNA viruses to complex eukaryotes, is, typically, close to the Eigen threshold, indicating that evolution solves an optimization problem with respect to replication fidelity, information content of the genome, and, possibly, variation (evolvability) [5].

**Figure 1:**
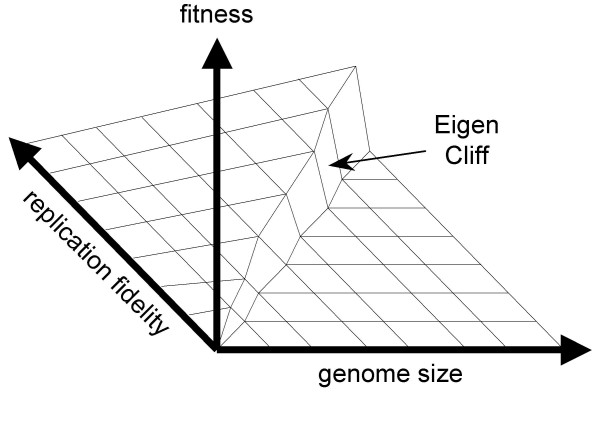
The Eigen threshold for replication fidelity. Fitness could potentially increase with the increase of the genome size and replication fidelity. However, exceeding the genome size limit, imposed by the fidelity that is attainable at the given point in evolution, leads to the "Error Catastrophe" [3], illustrated here as the "Eigen Cliff".

**Figure 2:**
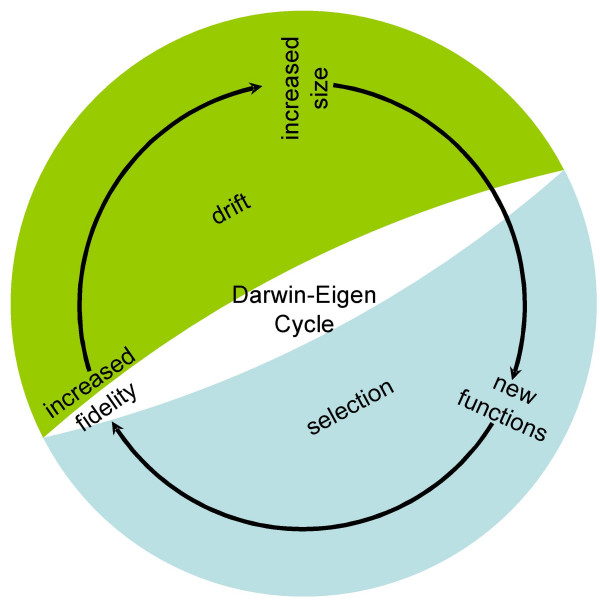
The Darwin-Eigen cycle. The Darwin-Eigen cycle, driven, in part, by selection and, in part, by drift, provides the path to the increasing complexity in course of the evolution of biological systems.

Taking the replication process over the Eigen threshold is required for sustainable replication and is, per force, a pre-requisite for the start of biological evolution (Fig. 1). Indeed, the very origin of the first organisms presents, at least, an appearance of a paradox because a certain minimum level of complexity is required to make self-replication possible at all, and high-fidelity replication requires additional functionalities that need even more information to be encoded. At the same time, the existing level of replication fidelity limits the amount of information that can be encoded in the genome [3,6,7]. What turns this seemingly vicious circle into the (seemingly) unending spiral of increasing complexity (the Darwin-Eigen cycle, after Penny [8]) is a combination of natural selection with genetic drift. Even small gains in replication fidelity are advantageous to the system, if only due to the decrease of the reproduction cost as a result of the increasing yield of viable copies of the genome. In itself, a larger genome is more of a liability than an advantage due to higher replication costs. However, moderate genome increase, e.g., by duplication of parts of the genome, or by recombination, can be fixed via genetic drift in small populations [9]. Replicators with a sufficiently high fidelity can take advantage of such randomly fixed and, initially, useless genetic material by evolving new functions, without falling off the "Eigen cliff" (Fig. 1). Among such newly evolved, fitness-increasing functions will be those that increase replication fidelity which, in turn, allows further increase in the amount of encoded information. And so the Darwin-Eigen cycle recapitulates itself in a spiral progression, leading to a steady increase in genome complexity (Fig. 2).

The crucial question on the origin of life is how did the Darwin-Eigen cycle start, i.e., how was the minimal complexity attained that is required to achieve the minimally acceptable replication fidelity. In even the simplest modern systems, such as RNA viruses with the replication fidelity of only ~10^-3^, replication is catalyzed by a complex protein replicase [10]. The replicase itself is produced by translation of the respective mRNA(s) which is mediated by a tremendously complex molecular machinery (see below). Hence the dramatic paradox of the origin of life: in order to attain the minimal complexity required for a biological system to get on the Darwin-Eigen spiral, a system of a far greater complexity appears to be required. How such a system could evolve, is a puzzle that defeats conventional evolutionary thinking, all of which is about biological systems moving along the spiral; the solution is bound to be unusual.

The origin of complex biological systems is a classical topic in evolutionary biology and, probably, the principal object of attacks of anti-darwinists of all ilk, including the notorious Intelligent Design movement. The gist of the criticisms is that many biological systems are not just complex but "irreducibly complex" and, as such, could never evolve via the Darwinian mechanism of gradual, stepwise adaptive change because intermediate stages of evolution would have no selective value and so could not be fixed. Darwin himself was perfectly aware of the problem and its dimensions and addressed it in one of the most famous passages of the *Origin*, the one on the evolution of the vertebrate eye [1]. The solution offered by Darwin and developed ever since in numerous works of evolutionary biology was straightforward in principle and extremely ingenious when it came to details. Darwin noticed that primitive eyes (or eye-like perceptive organs) were found in a variety of animals and outlined a hypothetical, multistage scenario for the evolution of the eye in which each simple, small step was selected for a particular advantage it conferred onto the evolving organism. Darwin depicted the gradual complexification of the organ of visual perception from a light-sensitive spot to a fully-fledged eye; in this example, the function of the organ, while evolving, remained, in principle, the same. When an evolutionary biologist strives to explain the origin of a truly novel system that is seen only in its elaborately complex state and, at face value, appears to be irreducibly complex, the task is much harder. Because evolution has no foresight, no system can evolve in anticipation of becoming useful once the requisite level of complexity is attained. Instead, the evolving system must have a selectable function(s) distinct from the modern one, a possibility recognized by Darwin [1] and emphasized by Gould in the concept of exaptation, that is, reassignment of function in the course of evolution [11,12]. In either case, the general Darwinian principle applies: evolution must proceed via consecutive, manageable steps, each one associated with a demonstrable increase in fitness. Darwin did not use a specific term for this crucial tenet of evolutionary biology; we will call it the ***Continuity Principle***, following the recent insightful discussion of this issue by Penny [8]. The developments in the 150 years since Darwin taught us to be more flexible about this principle than he was. It is no longer prudent to demand, as Darwin did, that all evolutionary changes are "infinitesimal"; some genome modifications may have had a substantial one time effect on fitness, e.g., those that involve horizontal gene transfer, gene loss, or genome rearrangement [13]. Furthermore, it cannot be demanded that every change is selectively advantageous because neutral or even slightly deleterious mutations can be fixed by drift, especially, in small populations [9,14]. Nevertheless, these newly discovered factors of evolution, however important by themselves, are but modifications of the Continuity Principle – evolution of complex systems still needs to be deconstructed into successive steps and explained in a Darwinian way.

We discussed the principles of evolution of complex biological systems at some length because they are most pertinent to the fundamental problem we wish to address here: the origin of the translation system and the genetic code. Indeed, the translation system might appear to be the epitome of irreducible complexity because, although some elaborations of this machinery could be readily explainable by incremental evolution, the emergence of the basic principle of translation is not. Indeed, we are unaware of translation being possible without the involvement of ribosomes, the complete sets of tRNA and aminoacyl-tRNA synthetases (aaRS), and (at least, for translation to occur at a reasonable rate and accuracy) several translation factors. In other words, staggering complexity is inherent even in the minimally functional translation system. Thus, as outlined above, it appears that the evolutionary origin of translation is to be sought along the exaptation route, i.e., by retrodiction of the ancestral functions of various components of the translation system that would allow them to evolve functionalities enabling their recruitment for translation.

Even this, however, does not do the full justice to the difficulty of the problem. The origin of translation appears to be truly unique among all innovations in the history of life in that it involves the invention of a basic and highly non-trivial molecular-biological principle, the encoding of amino acid sequences in the sequences of nucleic acid bases via the triplet code[15,16]. This principle, although simple and elegant once implemented, is not immediately dictated by any known physics or chemistry (unlike, say, the Watson-Crick complementarity) and seems to be the utmost innovation of biological evolution.

The obvious common wisdom is that a system as complex as the translation machinery, even in its primitive state (let alone the modern version, with its hundreds of RNA and protein components – see below), could not have emerged in one sweep. Such an abrupt emergence would appear an outright miracle and an obvious violation of the Continuity Principle. Elsewhere, one of us considers a different worldview that might bring the chance emergence of complex (pre)biological systems, in particular, translation and replication, within the realm of the possible [17]. Here, however, we address the formidable problem of the origins of translation within the Continuity Principle, by harnessing evidence from comparative analysis of the translation system components, theoretical and experimental work on the hypothetical primordial RNA world, and the experimental study of interactions between amino acids and their codons and anticodons. After synthesizing the evidence from all these lines of enquiry, we embark on evolutionary modeling, with its unavoidable element of speculation, in an attempt to construct a sequence of plausible, incremental stages each of which is associated with a selective advantage to the evolving pre-biological entities – in accordance with the Continuity Principle.

### Evolution of the translation system – the case for a complex RNA world

The design of the translation system in even the simplest modern cells (e.g., parasitic and endosymbiotic bacteria and archaea, such as *Carsonella*, *Mycoplasma*, or *Nanoarchaeon*) is extremely complex. At the heart of the system is the ribosome, a large complex of at least three RNA molecules and 60–80 proteins arranged in a precise spatial architecture and interacting with other components of the translation system in the most finely choreographed fashion [18-22]. These other essential components include the complete set of tRNAs for the 20 amino acids (~40 tRNA species considering the presence of isoacceptor tRNAs in all species), the set of 18–20 cognate aminoacyl-tRNA synthetases (aaRS), and a complement of at least 7–8 translation factors. An extraordinary feature of the translation system is the conservation of its core across all modern cellular life forms. Indeed, of all functional categories of proteins, translation is by far the most conserved one: among the ~60 proteins that are represented by an ortholog in every single cellular life form with a sequenced genome, over 50 are components of the translation machinery [23]. Together with the universal conservation of ~30 RNA species [three rRNAs, the signal recognition particle (SRP) RNA, and tRNAs of at least 18 specificities] and the virtual universality of the genetic code, this proves that, the substantial differences between the translation machineries of archaea (and the eukaryotic cytosol) and bacteria (and the eukaryotic organelles) notwithstanding, the modern translation system is the best preserved relic of the Last Common Universal Ancestor (LUCA) of modern cellular life forms. Put another way, the conservation of the core of the translation machinery is the strongest available evidence that some form of LUCA actually existed.

Given this extraordinary conservation of the translation system, comparison of orthologous sequences reveals very little, if anything, about its origins – because the emergence of the translation system is beyond the horizon of the comparison of extant life forms. Indeed, comparative-genomic reconstructions of the gene repertoire of LUCA point to a complex translation system including at least 18 of the 20 aaRS, several translation factors, at least 40 ribosomal proteins, and several enzymes involved in rRNA and tRNA modification; thus, it appears that the core of the translation system was already fully shaped in LUCA [24]. However, sequence and structure comparisons of protein and RNA components of the translation system itself are informative thanks to the extensive paralogy among the respective genes. Obviously, when the origin of each of a pair of paralogous genes antedates LUCA, the respective duplication must have been an even earlier event, so reconstruction of the scenario of such events opens a window into very early stages of evolution.

The story of the paralogous aaRS is particularly revealing. The aaRS form two distinct classes of 10 specificities each, with unrelated catalytic domains and distinct sets of accessory domains [25,26]. The catalytic domains of the class I and class II aaRS belong to the Rossmann fold and the biotin synthase fold, respectively. The analysis of the evolutionary histories of these protein folds has far-reaching implications for the early evolution of the translation system and beyond. It has been shown that the catalytic domains of the Class I aaRS form but a small twig in the evolutionary tree of the Rossmann fold proteins; the advent of the common ancestor of the aaRS is preceded by a number of nodes along the evolutionary path from the primitive, ancestral domain to the highly diversified state that corresponds to LUCA [27,28]. The striking corollary of this simple observation is that a substantial diversity of Rossmann fold domains has evolved prior to the series of duplications that led to the emergence of the aaRS of different specificities which, in turn, antedates LUCA. A very similar evolutionary pattern is implied by the analysis of the biotin synthase domain that gave rise to Class II aaRS [29]. Thus, even within these two folds alone, a remarkable structural and functional complexity had been attained before the fully-fledged RNA-protein machinery of translation resembling the modern one has evolved. The evolutionary analysis of the vast class of P-loop GTPases, in which a variety of translation factors comprise distinct, tight families, leads to essentially the same conclusions: in the succession of evolutionary bifurcations (tree branchings) that comprise the history of the GTPase domain, the translation factors are relatively late arrivals [30]; not to be forgotten that the GTPases are but one of the major branches of the P-loop fold [30]. This might strike one as counter-intuitive but it is an inevitable conclusion from the comparative analysis of ancient paralogous relationship between proteins within the translation system: with the interesting exception of the core ribosomal proteins, all proteins that play essential roles in modern translation are products of long and complex evolution of diverse protein domains. So here comes the Catch-22: for all this protein evolution to occur, an accurate and efficient translation system was required. This ancient translation system might not have been quite as accurate and efficient as the modern version but it will be a safe bet to infer that is must have been within an order of magnitude from the modern one in terms of fidelity and translation rates, to make protein evolution possible. However, from all we know about the modern translation system, this level of precision is unimaginable without a complex, dedicated protein apparatus [31].

Thus, the translation system presents us with the Darwin-Eigen paradox as clearly as it gets: for a modern-type, efficient and accurate translation system to function, many diverse proteins are needed, and for those proteins to evolve, a translation system almost as good as the modern one would be necessary. There is only one solution to this paradox, and it lies in an, at least, partial refutation of the first part of the above opposition: we are forced to conclude that a translation system comparable to the modern one in terms of accuracy and speed functioned without many proteins, possibly, without any proteins at all. Hence the very existence of a complex, elaborate RNA world (see the next section), in which a primitive version of the Darwin-Eigen cycle was already operating, can be conjectured from the comparative analysis of the translation system components (again, a different perspective on this issue is given elsewhere[17]).

This is not all the comparative analysis can do: comparison of RNAs themselves also yields important information and startling puzzles. The conservation of the structure, some sequence elements (e.g., the pseudouridine loop), and even modification sites of the tRNAs of all specificities (and, needless to say, all species) leaves no doubt that they all evolved from a single common ancestor [32-34]. Hence the second paradox of translation evolution ensuing from the comparison of modern sequences and structures: if, at some point in evolution, there was a single progenitor to tRNAs of all specificities, how could a translation system function – and, if there was no translation system at that stage, what would be the driving force of evolution of the amino-acid-specific tRNAs?

### Ribozymes and the RNA World

The famous central dogma of molecular biology [16] states that, in biological systems, information is transferred from DNA to protein through an RNA intermediate (the possibility of reverse information flow from RNA to DNA has been added after the discovery of reverse transcriptase):

DNA⇔RNA→protein

Obviously, when considering the origin of first life forms, one faces the proverbial chicken-and-egg problem: what came first, DNA or protein, the gene or the product? In that form, the problem might be outright unsolvable. Indeed, there is a crucial feedback in this system: to replicate and transcribe DNA, functionally active proteins are required, but production of these proteins requires accurate replication, transcription, and translation of nucleic acids. If one sticks to the triad of the Central Dogma, it is impossible to envisage what could serve as the starting material for the Darwin-Eigen cycle. Even removing DNA from the triad and postulating that the original genetic material consisted of RNA, while an important idea (see below), is not going to help much because the feedback remains as crucial as it is elusive. In order for evolution toward greater complexity to take off, the system needs to somehow get started on the Darwin-Eigen cycle prior to establishing this feedback.

The brilliantly ingenious and, perhaps, the only possible solution has been independently proposed by Woese [35], Crick [36], and Orgel [37] in 1967–68: neither the chicken nor the egg but what is in the middle, that is, RNA alone! The unique property of RNA that makes it a credible, indeed, apparently, the best candidate for the central role in the primordial replicating system is its ability to combine informational and catalytic functions. This notion has been greatly boosted by the study of ribozymes (RNA enzymes), which was pioneered by Cech and coworkers' discovery, in 1982, of the autocatalytic cleavage of the *Tetrahymena *rRNA intron [38], and by the demonstration, in 1983, by Altman and coworkers, that RNAse P is a ribozyme [39]. Since the time of these seminal discoveries, the study of ribozymes has evolved into a vast, expanding research area (at the time of this writing, March 1, 2007, the keyword 'ribozyme' retrieves 4883 documents from the PubMed database; for recent reviews, see [40-43]).

The discovery of ribozymes made the idea that the first replicating systems consisted solely of RNA molecules, which catalyzed their own replication, extremely attractive. In 1986, Gilbert coined the term "RNA world" to designate this hypothetical stage in life's evolution [44], and the idea caught up big way, becoming the leading, in fact, almost universally accepted hypothesis on the early stages of life's evolution [45-48].

The popularity of the RNA World hypothesis has, in turn, further stimulated ribozyme research, aimed, in large part, at testing the feasibility of various RNA-based catalytic activities, above all, perhaps, an RNA replicase. It is noteworthy that the main approach to developing ribozymes with desired activities is *in vitro *selection that, at least conceptually, mimics the Darwinian evolution of ribozymes thought to occur in the primeval RNA world [49,50]. Essentially, these directed selection experiments are designed in such a fashion that, from a random population of RNA sequences, only those are amplified that catalyze the desired reaction. In such multiple-round selection experiments, ribozymes have been evolved to catalyze an extremely broad variety of reactions. Since here we are concerned with the problem of the origin of translation, Table 1 lists only those of the ribozyme-catalyzed reactions that appear to be specifically relevant to this problem. Notably, all three elementary reactions required for translation, namely: i) amino acid activation through the formation of aminoacyl-AMP, ii) (t)RNA aminoacylation, and iii) transpeptidation (the peptidyltransferase reaction), have been successfully modeled with ribozymes (Table 1). It is particularly remarkable that the key self-aminoacylation reaction has been selected in vitro with considerable ease such that the best of the resulting ribozymes catalyzed it with a rate and specificity greater than those of the respective aaRS [51].

**Table 1:** Ribozyme activities relevant for the emergence of the translation machinery from the RNA world

**Reaction**	**Characteristics of the ribozyme**	**References**
Aminoacyl adenylate synthesis	Low efficiency formation of leucyl and phenylalanyl adenylates observed with a 114-nucleotide ribozyme.	[132]
Self-aminoacylation	Self-aminoacylation of a 43-nulceotide ribozyme with phenylalanine using phe-AMP as the substrate. A 77-nucleotide RNA catalyzed the same reaction with a specificity and aminoacylatin rate greater that those of PheRS.	[51, 146]
RNA 3'-aminoacylation In-trans	The smallest ribozyme capable of non-specific tRNA aminoacylation consists of 29 nucleotides. A 45-nucleotide ribozyme has been obtained with a broad spectrum of activity toward diverse tRNAs and amino acids. Larger ribozymes with highly specific and efficient aminoacylation activity reported.	[51, 147, 148]
*In vitro *selected peptidyltransferase ribozymes	Several ribozymes selected to form dipeptides from an amino acid esterified to AMP or a oligonucleotide and a free amino acid. Structural similarity observed between peptidyltransferase sibozymes and the relevant portion of 23S rRNA. Formation of Phe-Phe-tRNA reported for the 29-nucleotide aminoacylating ribozyme.	[128, 129, 149, 150]
Ribosomal peptidyltransferase	In the ribosomal large subunits, the peptidyltransferase center maps to an are containing only RNA, leading to the conclusion that the reaction is catalyzed by a ribozyme; however, identification of the active residues remains elusive.	[151–154]
Ribonucleotide polymerization	Ribozymes capable of extending a pre-annealed RNA primer by 10–14 nucleotides selected from a pool of RNA ligase ribozymes	[53, 54]

Understandably, major effort has focused on the demonstration of nucleotide polymerization and, ultimately, RNA replication catalyzed by ribozymes, the key processes for the hypothetical, primordial RNA World. While these reactions are not directly involved in translation, they are highly relevant to the problem considered here inasmuch as replication with a fidelity above the Eigen threshold is a pre-requisite of biological evolution (see above). The outcome of the experiments aimed at the creation of ribozyme replicases so far has been somewhat mixed. Ribozymes have been obtained capable of extending a primer annealed to a template by 10–14 nucleotides; initially, the ribozymes with this activity could function only by specific base-pairing to the template but, subsequently, general ribozyme polymerases of this class have been evolved through additional selection [52-56]. However, these ribozyme polymerases are still a far cry from processive, sufficiently accurate (in terms of the Eigen threshold) replicases, capable of catalyzing replication of exogenous templates and themselves, that appear to be a *conditio sine qua non *for the evolution of the hypothetical RNA World.

It is often noted that the RNA World is not just a concept supported by the catalytic prowess of ribozymes: while overshadowed by the multitude of proteins with catalytic and structural functions, the RNA World still lurks within modern life forms [57,58]. Reactions catalyzed by ribozymes, while by far less numerous than those catalyzed by protein enzymes, are of crucial importance in modern cells. The foremost case of a today's natural ribozyme is the ribosome itself, where the crucial peptidyltransferase reaction is catalyzed by large-subunit rRNA without direct participation of proteins [59-61]. In the nearly ubiquitous tRNA-processing enzyme RNAse P, the catalytic moiety is an RNA molecule whereas the protein subunits play the role of cofactors stabilizing the RNA catalyst and facilitating the reaction [62,63]. Furthermore, group I and group II self-splicing introns, which are widespread in bacteria and in plant, fungal, and protozoan organelles, are ribozymes that catalyze their own excision from RNA transcripts, often, facilitated by specific proteins, the maturases [64-69]. It is generally believed that the myriads of eukaryotic spliceosomal introns, as well as the snRNAs that comprise the active moieties of the eukaryotic spliceosomes, have evolved from Group II introns [68,69], leaving, perhaps, the most conspicuous imprint of the RNA World on modern genomes [70]. Similarly, in the smallest known infectious agents, viroids and virusoids, the ribozyme-catalyzed reactions are directly involved in replication: although the polymerization of nucleotides is catalyzed by a protein polymerase, processing of replication intermediates into genomic units depends on a built-in ribozyme [71]. The existence and importance of these (and, perhaps, other, still undiscovered) RNA-catalyzed reactions in modern cells imply a major role of RNA catalysts in the early evolution of life but in no way prove the reality of the primordial RNA world as it is defined above – a large community of RNAs possessing diverse catalytic activities and replicated by ribozyme polymerases. Nevertheless, these features of modern RNAs are fully compatible with such an evolutionary stage and greatly add to its plausibility. In particular, the fundamental fact that the peptidyltransferase reaction in the ribosome is catalyzed by a ribozyme strongly suggests that this was the functional mode of the primordial translation system.

To recapitulate, three independent lines of evidence converge in support of a major role of RNA, and in particular, RNA catalysis at the earliest stages of life's history, and are compatible with the reality of a complex, ancient RNA world that was first postulated by Woese, Crick, and Orgel on purely logical grounds. First, comparative analysis of the protein components of the translation machinery and their homologs involved in other functions strongly suggests that extensive diversification of the protein world took place at the time when the translation system was comprised, primarily, of RNA. Second, several classes of ribozymes operate within modern cells, and their properties are compatible with the notion that they are relicts of the ancient RNA world. Third, while limited in scope and, obviously, inferior in catalytic activity compared to protein enzymes [41], ribozymes have been shown or, more to the point, evolved to catalyze a remarkable variety of reactions including those that are central to the evolution of translation (Table 1).

All these arguments in favor of the reality of the RNA World notwithstanding, there are two major sources of doubts. First, despite all invested effort, the *in vitro *evolved ribozymes remain (relatively) poor catalysts; the lack of efficient ribozyme polymerases seems particularly troubling. Admittedly, it might be unrealistic to expect that experiments on *in vitro *evolution of ribozymes could easily mimic the actual complexity of the primordial RNA world. Indeed, although these experiments harness the power of selection, they are, obviously, performed on a totally different time scale and conditions that cannot possibly reproduce those of life's origin. The latter, of course, are not known but it seems reasonable to surmise that, if there was a complex RNA World at the brink of the Translation Breakthrough, it was brought about by millions of years of evolution of ensembles of replicating RNAs in a compartmentalized environment similar, at least, in principle, to the networks of iron sulfide compartments existing at hydrothermal vents [72-74]. The environment of this type can be reproduced in the laboratory but condensing eons of evolution into a manageable timescale is a grand challenge. Interestingly, a recent simulation study indicates that, if there was some RNA synthesis in such compartments[75,76], the resulting polyribonucleotides would accumulate to very high concentrations, an observation that increases the plausibility of this model. Of course, this scenario remains a model; other forms of compartmentalization are conceivable.

A recent study of Szathmary and coworkers puts some important numbers on the complexity that, potentially, might be attainable in the RNA World and the replication fidelity required to reach this level of complexity [77]. An estimate based on the functional tolerance of well-characterized ribozymes to mutations suggests that, at a fidelity of 10^-3 ^errors per nucleotide per replicase cycle, an RNA "organism" with ~100 "genes" the size of a tRNA (~80 nucleotides) would be sustainable. This level of fidelity would require only an order of magnitude improvement over the most accurate ribozyme polymerases obtained by *in vitro *selection [52,78]. Conceivably, this is, roughly, the intrinsic complexity limit on ensembles of co-evolving "selfish cooperators" that might have been the "organisms" of the RNA world [74]. As aptly commented by Poole, "Getting from an RNA world to modern cells just got a little easier" [79]. Of course, "a little" is a crucial qualification here as all this evidence falls far short from proving the reality of a fully fledged RNA world; nevertheless, in the rest of this article, we proceed with the RNA world as a premise.

Even under the best case scenario, the RNA world does not appear to have potential to evolve beyond very simple "organisms". To attain greater complexity, invention of translation and the Protein Breakthrough were required. However, the selective forces underlying the emergence of the translation system in the RNA World remain obscure, and tracing the path to translation is extremely hard. This lack of clarity with regard to the continuity of evolution from the RNA World to an RNA-protein world can be construed as a second major objection against the RNA World as a crucial stage of life's evolution, an objection, perhaps, even more prohibitive than the first one, dealing with the imperfection of ribozymes. A radical alternative, "no RNA World" hypothesis, is considered elsewhere [17]. In the rest of this article, we discuss possible ways to derive the translation from the RNA World through a path of evolution adhering to the Continuity Principle.

### The nature and origins of the genetic code: a stereochemical correspondence between amino acids and codons or anticodons, a frozen accident, selection, or all of the above?

To understand how translation might have emerged, the nature and origin of the codon assignments in the universal genetic code are crucial. The problem of code evolution fascinated researchers even before the code was fully deciphered, and the earliest treatises on the subject already clearly recognized three, not necessarily mutually exclusive models: i) steric complementarity resulting in specific interactions between amino acids and the cognate codon (codon recognition model, or CRM) or anticodon triplets (anticodon recognition model, or ARM), ii) "frozen accident" – fixation of a random code that would have been virtually impossible to significantly change afterwards (frozen accident model, or FAM), and iii) adaptive evolution of the code starting from an initially random codon assignment [35,36,80-86]. The internal structure of the code is such that codons for related amino acids are adjacent in the code table resulting in a high (although not maximum) robustness of the code to mutations and translation errors as first noticed by Woese at a qualitative level [35,82] and subsequently demonstrated quantitatively [87-93]. The robustness of the code seems to falsify the frozen-accident scenario in its pure form; however, the stereochemical model, the selection model, a combination thereof, or frozen accident followed by adaptation all could explain the observed properties of the code.

The principal dilemma is whether or not a stereochemical correspondence between amino acids and cognate triplets (in the form of either CRM or ARM) exists or not. The answer to this straightforward question proved to be surprisingly elusive. The early attempts to establish specificity in interactions of (poly)amino acids and polynucleotides have been inconclusive, indicating that, if a correspondence exists, it must be much less than precise, and the interactions involved would be weak and dependent on extraneous factors [94-96]. Although some tantalizing cases of non-randomness in amino-acid-nucleotide interactions have been claimed (e.g., [97-102]), one is forced to conclude that, in general, the attempts to demonstrate such interactions directly have failed.

A recent resurgence of the stereochemical hypothesis was brought about by the application of the selection amplification (SELEX) methodology for isolation of oligonucleotides (aptamers) that specifically bind amino acids [103,104]. The latest survey by Yarus and coworkers reports detailed aptamer data for 8 amino acids: phenylalanine, isoleucine, leucine, histidine, glutamine, arginine, tyrosine, and tryptophan [104]. With the sole exception of glutamine, the aptamers for each amino acids were enriched for codon and/or anticodon triplets at a statistically highly significant level [104-106]. On the whole, associations with anticodons were more pronounced than those with codons. However, the results are complementary in that arginine (the amino acid characterized in greatest detail in aptamer experiments) showed a significant enrichment only for codons in binding sites, whereas for phenylalanine, leucine, and tryptophan, the binding sites were significantly enriched for anticodons; rather surprisingly, isoleucine and tyrosine were associated with both types of cognate triplets [104]. Taken together, the experimental results on aptamer binding that, in the case of arginine, have been analyzed in great detail for possible effects of statistical and chemical artifacts [107] are construed as a strong argument for the stereochemical hypothesis of code origin [104]. Moreover, for histidine, isoleucine, and tryptophan, it has been shown directly that the simplest binding aptamers contained the cognate codon or anticodon [108-112], lending credence to the idea that similar molecules might be relevant for modeling evolution in the RNA world [104].

Nevertheless, serious questions remain as to the ultimate validity and relevance of these results. The presence of both codons and antidocons in aptamers binding several amino acids is hard to interpret in terms of stereochemical complementarity. Furthermore, the amino acids for which detailed aptamer data is available are those that have complex side chains (which, presumably, mediate interactions with the aptamers) and are thought to be late recruitments to the genetic code [113]. At least, until similar results are obtained for simpler, supposedly, ancient amino acids, it is hard to view the aptamer selection results as a definitive case for the stereochemical hypothesis of code origin.

A different, and elegant version of the stereochemical correspondence hypothesis has been proposed by Copley and coworkers[114]. This scenario links the origin of the code to the synthesis of amino acids by postulating that, under prebiotic conditions, dinucleotides covalently bound α-keto acids and specifically enhanced amino acid synthesis from these precursors. Unfortunately, there is no empirical evidence in support of this interesting model.

Thus, the jury is still out with regard to any role direct interactions between amino acids and cognate triplets might have played in the origin of the code. Accordingly, in what follows, we strive to be objective and consider the origin of the code in three distinct contexts: i) specific interaction between amino acids and the cognate codons (CRM), ii) specific interactions between amino acids and the cognate anticodons (ARM), and iii) frozen accident (FAM) as the starting point for the evolution of the code.

### Previous hypotheses on the origin of translation

During the 40 years since the discovery of the translation mechanism and deciphering of the genetic code, numerous theoretical (inevitably, speculative, sometimes, far fetched, often, extremely ingenious) models of the origin and evolution of various components of the translation apparatus and aspects of the process itself have been proposed. A comprehensive, critical review of this literature would be a truly daunting task and will not be attempted here. We outline only a few of the more straightforward and, in our opinion, more plausible, evolutionary schemes and then discuss in somewhat greater detail the only published coherent scenario for the evolution of the translation system we are aware of.

One popular and potentially important idea on the origin of the genetic code is the hypothesis of Szathmary on the role of so-called coding coenzyme handles (CCH), i.e., oligonucleotides with various ribozyme activities using amino acids as cofactors, as evolutionary progenitors of tRNAs [115-117]. This hypothesis ties in with the idea that tRNAs evolved by two successive duplications of amino-acid-binding hairpins [118]. The CCH are thought to have assembled via their proto-anticodons on emerging mRNAs. A modification of the CCH hypothesis proposed by Knight and Landweber involves evolution of aminoacylating ribozymes (which is compatible with the available experimental data – see Table 1) and emergence of non-templated, ribozyme-mediate peptide synthesis as an intermediate stage in the evolution of translation [107]. An alternative to the CCH scheme is the direct-RNA-templating (DRT) hypothesis of translation origin proposed by Yarus [119]. Under the DRT model, the original form of amino-acid-proto-tRNA interaction was direct binding, presumably, via anticodon triplets; subsequently, direct binding has been supplanted by the adaptor mechanism, probably, with the participation of aminoacylating ribozymes, as under the modified CCH hypothesis.

These and other hypotheses tackle important aspects of the origin and evolution of the translation system. However, they all stop short of proposing a complete, coherent scenario for the transition from the RNA world to the modern mode of translation. We believe that the reason for the near lack of such scenarios in the current literature is the formidable difficulty of breaking this transition into incremental steps associated with a biologically plausible selective advantage, thus making the entire transition compatible with the Continuity Principle.

We are aware of two proposals that come closest to such a complete scenario, and it seems to be more than a remarkable coincidence that the two present essentially the same model, differences in detail notwithstanding. The essence of this model, originally sketched by Altstein [120-122], and later, independently and more completely developed by Poole, Jeffares, and Penny [8,123], is that the ribosome and the translation mechanism are derived from an ancient ribozyme replicase.

Let us examine in some detail the model of Poole and coworkers, which is better reconciled with various facets of the RNA World than the original proposal of Altstein (not surprisingly, given that the first version of Altstein's hypothesis [120] has been proposed prior to the discovery of ribozymes). Crucially, in this model, the protoribosome is postulated to have functioned as a "triplicase", i.e., a complex ribozyme combining the activities of a RNA polymerase and a RNA ligase by building a nascent RNA molecule complementary to the template in three-nucleotide steps. The "triplicase"-protoribosome would facilitate the assembly of tRNA-like molecules (perhaps, analogous to the CCH) on the template RNA through base-pairing of (proto)anticodons with complementary triplets (codons) on the template, cleaving off the rest of the pre-tRNA, and joining (ligating) adjacent triplets (Fig. 2 in [8,123]). A RNA-based replication mechanism involving complementary interaction of trinucleotides with the template, as opposed to mononucleotides, was deemed plausible by Poole et al., given the low efficiency (long characteristic turnover times) of ribozymes. A complex of template RNA with a complementary trinucleotide would persist orders of magnitude longer than a complex with a mononucleotide, giving the triplicase a chance to ligate the adjacent triplets. The hypothetical triplicase mechanism was considered particularly plausible [8] in view of the demonstration, by Fredrick and Noller, that the ribosome, without the involvement of translation factors, threads mRNA through the ribosome in three-nucleotides steps, with concordant movements of tRNAs [124]. Thus, the modern ribosome, of which the primary functional part is rRNA, is a versatile machine that catalyzes the stepwise joining of amino acids to form polypeptide chains and also mediates the associated movements of RNA molecules. It seems tempting to view this mechanism, which is crucial for modern translation, as a relic of the primordial "triplicase" system of RNA replication [8].

Of course, the transition from a triplicase to a modern-type translation-replication system requires the emergence of the genetic code, in this case, at the level of amino acid recognition by the proto-tRNAs, and the feedback between translation and RNA replication. Furthermore, a subfunctionalization stage would be required where the triplicase would give rise to separate proto-ribosome and replicase, the latter having to switch from triplet joining to the conventional, one nucleotide at a time, replication mechanism. Perhaps, most damningly, the triplicase/protoribosome would have to be a tremendously advanced, complex RNA machine. Poole et al. [123] are not particularly specific about the organization of this machine and the likely mechanisms of and selective forces behind each of the necessary evolutionary steps, which renders the triplicase model incomplete and leaves one with the suspicion that, all its attraction notwithstanding, the triplicase might not be the most likely solution to the origin of translation problem. Nevertheless, regardless of the validity of its details, the triplicase model drives home a crucial point: evolution having no foresight, protein synthesis could not be the selective advantage that fuelled the initial evolution of the translation system; inevitably, it must have evolved via the exaptation route.

An overview of the existing models for the origins of translation and coding shows that none of them, not even the attractive triplicase model, offer a complete, compatible with the Continuity Principle outline of the path to the Protein Breakthrough. In the rest of this article, we explore three versions of such scenarios, two building upon specific interactions between amino acids and codons or anticodons, respectively, and the third one centered around frozen accident. We draw on aspects of the previously published models, in particular, the DRT, CCH, and triplicase hypotheses, and the experimental data on ribozymes, and also propose several original steps.

## A conceptual scenario for the origin of translation and the genetic code

### The assumptions, premises, and settings

**Figure 3:**
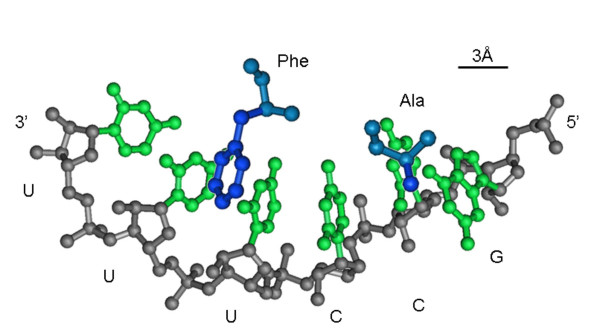
A crude stereochemical model of a direct interaction of two amino acid with a hexanucleotide representing their cognate codons.

1. ***The Continuity Principle ***remains the central principle of evolution despite the demonstration of the importance of fixation of neutral or slightly deleterious changes due to drift, and the possibility of substantial single-step innovations brought about by HGT, recombination, duplication, and other processes. All these important phenomena are but additions that only emphasize the basic validity of the Continuity Principle: evolution has no foresight and does not perform miracles. It proceeds step-by-step, and each step is, generally, associated with a selective advantage for the bearers of the respective innovation, even as some of these steps might not be infinitesimal as Darwin thought they had to be.

2. ***A diverse RNA world antedating translation*. **As discussed above, the latest results on the catalytic activities of ribozymes suggest the possibility of a versatile RNA world that already harbored a considerable diversity of catalytic activities, including, among others, RNA polymerases (replicases). Comparative analysis of translation system components points in the same direction, i.e., indicates that the primordial translation system consisted (predominantly) of RNA. The RNA World is a conjecture not a proven fact but, for the purpose of this paper, we assume that it existed.

3. ***Evolution has no foresight ***– thus, before there were functional proteins facilitating replication, production of proteins could not be the driving force behind the evolution of the translation system. Translation must have evolved as a by-product of selection for some other function, i.e., via the exaptation route.

4. ***Fidelity of translation in the late RNA world was comparable to that of the modern translation*. **Counter-intuitively but undeniably, the fidelity of the primitive translation system that evolved within the ancient RNA world could not have been dramatically lower than that of the modern translation system, with all its numerous, essential proteins. This is the logical conclusion from the results of protein sequence and structure comparisons which reveal extensive diversification of at least several protein folds antedating the emergence of the protein components of the modern translation system (in principle, it is possible to imagine that the primordial translation system included a complement of proteins distinct from the modern one; however, this hypothesis not only has no empirical support but also leads to infinite regression). A corollary is that, already within the confines of the RNA world, the translation machinery, in its principal features, resembled the modern one. In particular, it is impossible to imagine a high-fidelity translation system functioning without a set of tRNAs for many, probably, most of the 20 amino acids found in modern proteins.

5. ***Specific interactions (or lack thereof) between amino acids and codons or anticodons***. We believe that the jury is still out on the reality and relevance of putative specific interactions between amino acids and cognate triplets – either codons or anticodons. Accordingly, we formulate and explore three alternative models for translation origin depending on whether or not amino acids specifically recognize cognate triplets: i) interaction of amino acids with codons (CRM), ii) interaction of amino acids with anticodons (ARM), and iii) no specific interactions between amino acids and any of the cognate triplets – the frozen-accident model (FAM).

6. ***Adaptors must have been part of the emerging translation system from the start because peptide formation via direct binding of amino acids to a RNA template is stereochemically implausible***. Indeed, a simple 3D model shows that, even if there was a specific affinity between amino acids and cognate codons, the distances between amino acid assembled on an RNA template would be far too long for the formation of peptide bonds (Fig. 3). Thus, any direct recognition of amino acids by cognate triplets or other sites must have occurred at the level of the adaptors from the earliest stages of the evolution of the translation system. In that regard, it is highly encouraging that highly efficient self-aminoacylating ribozymes are among thos that are most easily selected *in vitro *[51].

7. ***Ensembles of selfish cooperators – genetic elements co-existing in a compartmentalized habitat***. The models detailed in the next section depend on the existence of a certain level of complexity in the RNA world – manifested not only in the diversity of catalytic activities but also in the existence of co-selected ensembles of replicating RNA molecules, the "selfish cooperators" [74]. The notion of selfish cooperators, related to the previously developed stochastic corrector model [125,126], entails co-existing, functionally coupled molecules (e.g., replicases and ribozymes that catalyze the synthesis of RNA precursors) that are physically confined (compartmentalized) and selected as a group. We are considering selfish cooperators within the framework of a particular scenario of the early evolution of life that implicates networks of inorganic compartments, existing at hydrothermal vents on the ocean floor and consisting, primarily, of iron sulfide, as the hatcheries of pre-cellular life [73,74]. The models developed here are not, actually, linked to this particular scenario, which we adapt for the sake of concreteness; however, co-selected ensembles of RNA molecules and some form of compartmentalization are salient conditions.

8. ***Extensive formation of non-templated peptides in ribozyme-catalyzed reactions occurring within the compartments ***and stimulation of various ribozymes by peptides- an optional but plausible condition that would boost the model developed here. Abiogenic synthesis of at least several amino acids occurs readily in numerous variations of the classical Miller experiment and, more notably, ribozymes have been selected that efficiently catalyze non-templated synthesis of diverse peptides [127-129].

### The model: emergence of the translation system in the RNA world

Despite substantial differences caused by the nature of amino-acid-triplet interactions incorporated into the model (or no interactions at all), the three models – CRM, ARM, and FAM – have many features and steps in common. As we will point out, it seems that these steps are, in effect, logically inevitable in any model of the evolutionary origin of translation. Therefore, in the presentation of these models that follows, the common steps are outlined just once, and forking paths are taken consecutively as they emerge (the designations of the model-specific steps have suffixes CRM, ARM, and FAM).

**Figure 4:**
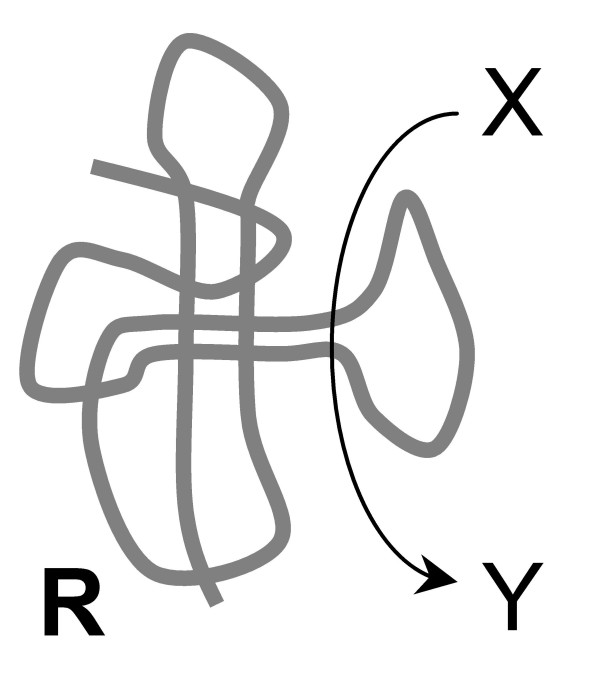
Origin of the translation system and the genetic code in the RNA World. Step 0 (the pre-requisite): a ribozyme **R **catalyzes an arbitrary reaction beneficial for an ensemble of selfish cooperators.

As outlined above, the starting point of all scenarios for the origin of translation is a replicating ensemble of selfish cooperators consisting of RNA molecules with various ribozyme activities and existing within a network of inorganic compartments [73,74]. One of the functions performed by these ribozymes is that of a replicase; other activities, such as RNA precursor synthesis, are likely to be present as well (Fig. 4). The scenarios include the following steps.

**Figure 5:**
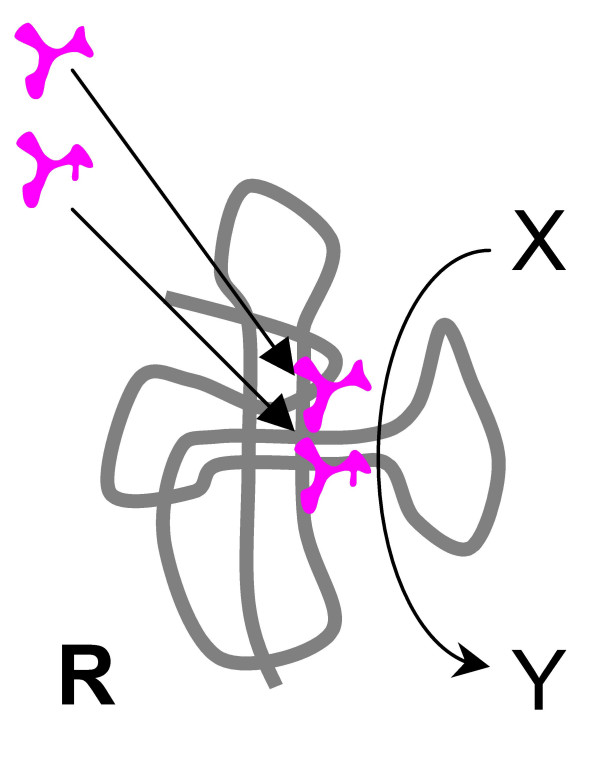
Origin of the translation system and the genetic code in the RNA World. Step 1: amino acids stimulate the activity of the ribozyme **R**.

**Figure 6:**
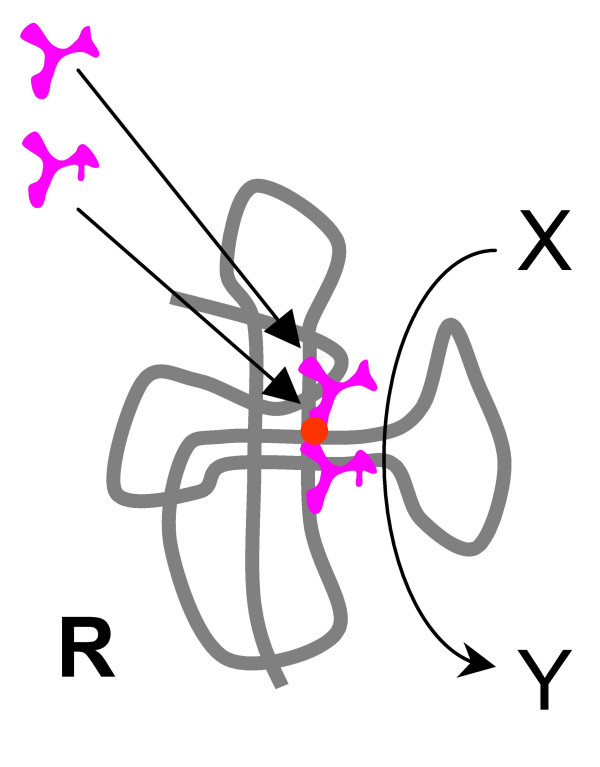
Origin of the translation system and the genetic code in the RNA World. Step 2: the ribozyme **R **evolves an additional enzymatic activity, that of a peptide ligase; enhanced stimulation of the original reaction by the synthesized peptide ensues. One of the joined substrates is likely to be an activated amino acid derivative, such as an aminoacyl adenylate (see text).

0. Ribozyme **R **(Fig. 4) is a part of an ensemble of selfish cooperators within a compartment. This ribozyme should possess sufficient complexity to catalyze the reaction (X→Y) affecting the fitness of the ensemble and to include a certain number of evolvable positions allowing, in principle, the emergence of new activities.

1. Two or more abiogenic amino acids present in the compartment bind to **R**. Specific binding of the amino acids is mediated by an *ad hoc *binding site present in **R**. Involvement of a stereochemical proto-code (in the form of either ARM or CRM) at this stage is possible but would not substantially affect the proposed scenario. It is postulated that the bound amino acids enhance the X→Y reaction catalyzed by **R **(Fig. 5). As indicated above, ribozymes strongly stimulated by peptides have been produced by *in vitro *selection [130]. Conceivably, stimulation of **R **by amino acids would be picked up by natural selection, in the context of the selfish cooperative evolution, leading to gradual perfection of the spatial alignment of amino acids on **R **and selection of the optimal sequence and structure for amino-acid-binding.

2. **R **evolves an additional peptide ligase activity, yielding oligopeptide **P **from adjacent amino acids bound to **R **(Fig. 6). Highly active ribozymes with peptide ligase activity, albeit with low specificity, have been obtained by *in vitro *selection [127,131]. It is most likely, however, that only short peptides consisting of, at best, four-five amino acids, could be synthesized by this class of ribozymes. The selective advantage of this innovation would be the increased stability of the reactive complex resulting in a further boost to the X→Y reaction.

An inevitable question with regard to this step is where does the energy required for the peptide bond formation come from. In the case of experimentally characterized ribozyme peptide ligases, one of the substrates is an aminoacyl adenylate, so the energy of the ester bond is utilized [127,131]. This mimics the situation in translation where the aminoacyl adenylate is used by the aaRS to charge the cognate tRNAs, and the high-energy ester bond of the latter is utilized for transpeptidation. It is not inconceivable that the primordial peptide ligase functioned in the same mode using aminoacyl adenylates or other activated derivatives of amino acids produced by other ribozymes; indeed, ribozymes that catalyze this reaction have been reported [132].

**Figure 7:**
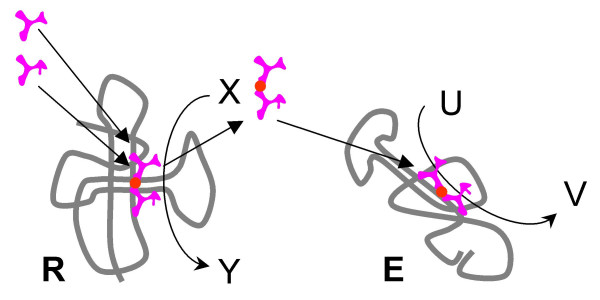
Origin of the translation system and the genetic code in the RNA World. Step 3: a peptide with generic ribozyme-stimulating properties is released from the ribozyme **R **and stimulates the activity of a distinct ribozyme **E**.

**Figure 8:**
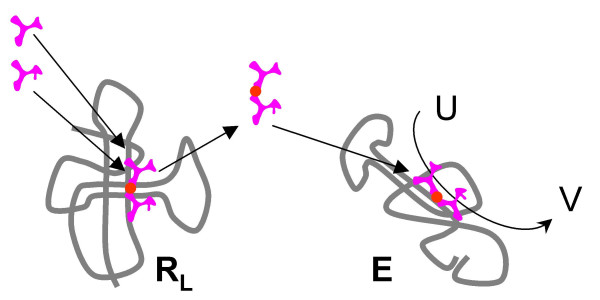
Origin of the translation system and the genetic code in the RNA World. Step 4: the original activity of the ribozyme **R **(X-Y) and the peptide ligase activity are apportioned between two ribozymes as the result of duplication and subfunctionalization; the ancestor of the large ribosomal subunit (**R**_**L**_) emerges.

**Figure 9:**
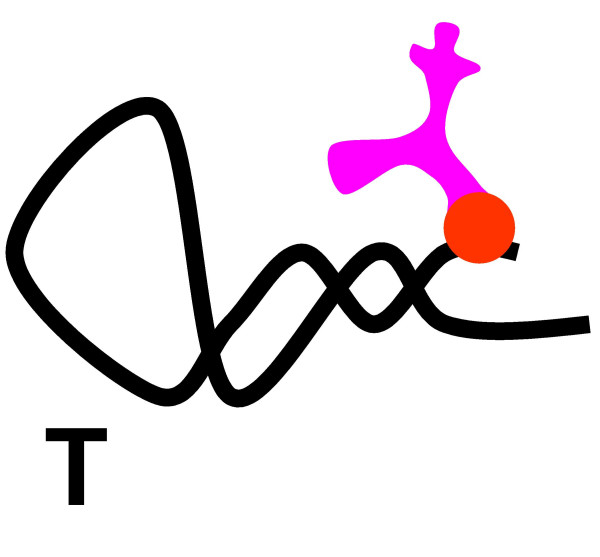
Origin of the translation system and the genetic code in the RNA World. Step 5: Small amino-acid-binding RNAs (**T **RNAs) evolve via selection for accumulation of amino acids. Once species of the **T **RNAs evolves the capacity of autocatalytic aminoacylation, further enhancing amino acid accumulation, The actual substrate, probably, was an activated amino acid derivative, such as an aminoacyl adenylate.

3. Spontaneous disassembly or decay of **R **would release the peptide **P **into the compartment (Fig. 7). If **P **has a generic ribozyme-stimulating and/or ribozyme-stabilizing capacity, it might be captured by another ribozyme **E**, which catalyzes a different reaction (U→V). An interesting example could be a peptide containing a pair of acidic amino acids and coordinating a magnesium ion as seen in a great variety of unrelated modern nucleic acid metabolism enzymes (polymerases, nucleases, ligases, topoisomerases, and more) [133-135]. If **P **boosts the catalytic activity of **E**, it again increases the fitness of the entire ensemble. Consequently, selection will favor appropriate adjustments to **E**, strengthening its association with **P**, and further increase of the selective pressure on **R **to keep producing **P**.

4. With the activity of **E **relying on the presence of **P**, a copy of **R **(**R**_**L**_) might lose the original X→Y activity, with a concomitant enhancement of the amino acid ligase activity, whereas other copies (**R**_**0**_) would retain the original activity, still enhanced by the peptide **P **(Fig. 8). This would be a typical case of subfunctionalization that is thought to be the preferred route of evolution of genes after duplication in modern genomes [136,137]. Subfunctionalization might have been important already in the RNA world, with the benefit of improved catalysis by **R**_**0 **_and **E **outweighing the increased replication cost (the necessity to maintain both **R**_**L **_and **R**_**0**_).

5. Widespread peptide-assisted catalysis in the compartmentalized pre-biological system makes amino acids a useful commodity for the evolving selfish cooperatives. Amino acids are small polar molecules that easily diffuse, especially, through the Fe-S gel, the principal constituent of the hydrothermal compartments [72,73]. Accumulation of amino acids (along with other important molecules) within a compartment, obviously, would be beneficial. Small, amino-acid-binding RNAs (**T**) evolve under the pressure of selection for amion acid accumulation ; these molecules would be analogous to amino-acid-binding aptamers that have been studied in a variety of experiments [103,104,110]. Originally, the T RNAs bind amino acids non-specifically. Autocatalytic aminoacylation of the 3' end of RNA **T **evolves, resulting in an increase in affinity and specificity of amino acid binding (Fig. 9). As with the peptide ligase in step 2, there should be a source of energy for this reaction; activated amino acid derivatives, such as aminoacyl adenylates would serve in this capacity. Since the aminoacyl-RNA bond is a high-energy one, the association between RNA **T **and the amino acid remains labile like in modern aminoacyl-tRNAs (e.g., Ref. [138], p. 57).

6. Different species of **T **RNAs specifically binding different amino acids evolve by duplication and diversification, with the retention of variants driven by selection for efficient accumulation of a broad repertoire of amino acids.

**Figure 10:**
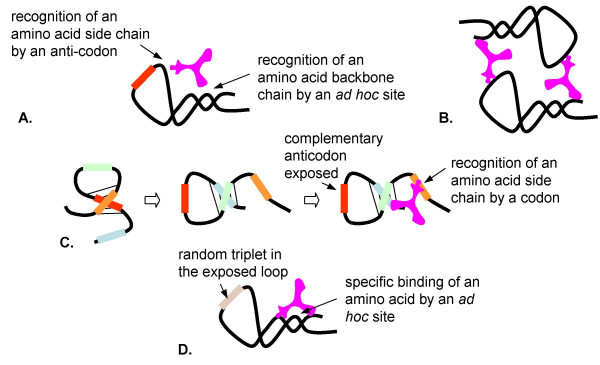
Origin of the translation system and the genetic code in the RNA World. Step 6: Amino-acid-specific variants of RNA **T **evolve by duplication and subfunctionalization. The specifics of **T **RNA-amino acid interaction depend on the mode amino acid recognition, CRM, ARM, or FAM. A. ARM: recognition of the amino acid residue by the anticodon loop and of the amino acid backbone by the stem of the RNA **T**. B. ARM: formation of dimers facilitates the stereochemically unhindered binding of amino acids. C. CRM: RNA **T **exists in two alternative folding conformations. In one conformation, the codon is complementary paired with the anticodon; in the other confirmation, the codon binds a cognate amino acid, and the anticodon is exposed. D. FAM: the amino acid is recognized by an "ad hoc" site unrelated to the codon or the anticodon.

The specific details of the binding mechanism would differ under the different modes of the amino acid-RNA recognition. Under the ARM, binding of the amino acid by the cognate RNA **T **would be mediated, in a concerted fashion, by two distinct moieties, the amino-acid-specific anticodon loop and the stem region with a non-specific affinity to the amino acid backbone (NH_3_-C^α^-COOH) (Fig. 10). The sterical problem of binding a small amino acid molecule by two separate regions of RNA **T **could be solved either by tight folding of the RNA chain or by the formation of dimers where a pair of RNA **T **molecules binds two amino acids (Fig. 10).

The CRM would require a similar but more complicated binding mechanism. Since, ultimately, the anticodon must be left exposed in a mature RNA **T**, one can envisage a folding flip between two conformations (one of them involving a complementary pairing of codon and anticodon), induced by the interaction with the cognate amino acid (Fig. 9).

Finally, FAM would require a different mode of amino acid recognition by RNA **T **whereby the recognition site is unrelated to either the codon or the anticodon, whereas the sequence of the exposed loop (the ancestor of the anticodon loop) in RNA **T **is chosen by chance (Fig. 10).

Regardless of the specific model (even under FAM), this is the critical step where the correspondence between amino acids and cognate triplets is established, directly or indirectly, creating the basis of the genetic code.

**Figure 11:**
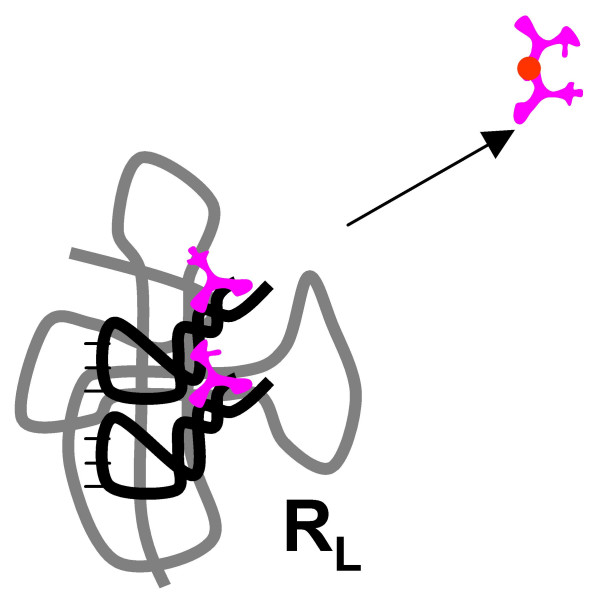
Origin of the translation system and the genetic code in the RNA World. Step 7: The proto-large subunit, **R**_**L**_, evolves the capacity to bind aminoacyl-**T **RNAs, resulting in more precise amino acid positioning on **R**_**L**_. The activity of **R**_**L **_switches from amino acid ligation to transpeptidation, resulting in an increased peptide yield.

**Figure 12:**
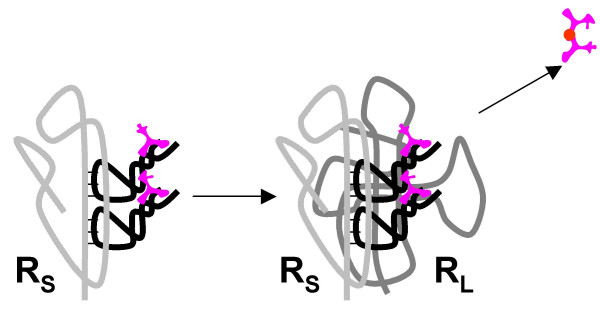
Origin of the translation system and the genetic code in the RNA World. Step 8: An accessory RNA subunit, **R**_**S **_(progenitor of the small ribosomal subunit), capable of binding aminoacyl-T RNAs through interaction between complementary base triplets.

7. Ribozyme **R**_**L **_evolves the capacity to bind aminoacyl-**T **RNA complexes instead of individual amino acids (Fig. 11). This would to result in an improvement in the stability and spatial precision of binding. The primary biochemical activity of **R**_**L **_changes from amino acid ligation to transpeptidation, which would result in an increased yield of peptides thanks to the high energy of the aminoacyl-RNA bond. Notably, it has been shown that the 50 S subunit of bacterial ribosomes, of which the ribozyme **R**_**L **_is deemed the progenitor, is capable of catalyzing the transpeptidation reaction at a rate comparable to that displayed by the complete ribosome [139]. These findings seem to support the plausibility of this step of the model.

8. An accessory RNA subunit **R**_**S **_evolves, driven by selection for increasingly efficient binding and positioning of aminoacyl-**T **complex on **R**_**L **_(Fig. 12). The burden of specific recognition shifts from an *ad hoc *(Van der Vaals and hydrogen-bonds) interaction between RNA **T **and **R**_**L **_to the specific base-pairing between the exposed (now anticodon) loop of **T **and an extended RNA strand of **R**_**S**_. The transpeptidation activity remains the function of **R**_**L**_. This is the crucial step of the emergence of *bona fide *translation, a mechanism based on the adaptors (proto-tRNAs, the **T **RNAs in this model) combining amino acids with the cognate codons [15].

The evolutionary path from the set of primitive T RNAs (Fig. 10) to the modern tRNAs seems mysterious given the indisputable common ancestry of tRNAs of all specificities (see above). Conceivably, at the early stages of the transalation system evolution outlined in steps 1–8, different species of **T **RNAs evolved along, roughly, parallel (convergent) paths. However, the common origin of tRNAs implies a subsequent bottleneck through which only a single winner has passed, an L-shaped molecule with the acceptor CCA 3'-end. Selection for spatial complemtarity and efficient interaction between the aminoacylated **T **RNAs and the peptidyl-transferase **R**_**L **_could be the driving force behind the selection for this structure. This selection originally would affect only one **T **RNA, perhaps, the one chargeable with the most abundant primordial amino acid. Since a relatively minor modification (a concerted change in the amino-acid-binding site and the anticodon loop) would switch the specificity of the proto-tRNA, a sweep by a single proto-tRNA species, taking over the function of other, unrelated and unevolved, **T **RNAs one by one, seems to be plausible. We tentatively place this sweep in an early stage in the evolution of the translation system; however, an alternative possibility is that it took place at a later stage, concomitantly with the evolution of aaRS and their takeover of the key role in the pairing of amino acids with the cognate anticodons.

**Figure 13:**
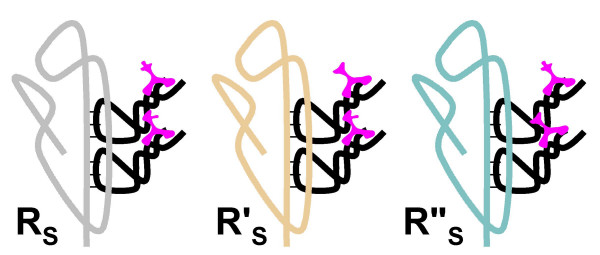
Origin of the translation system and the genetic code in the RNA World. Step 9: Amino-acid-specific variants of **R**_**S **_evolve by duplication and subfunctionalization.

**Figure 14:**
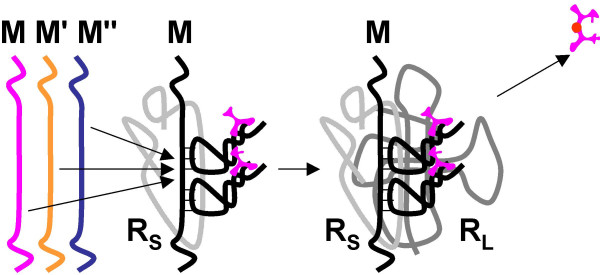
Origin of the translation system and the genetic code in the RNA World. Step 10: The proto-small subunit, **R**_**S**_, evolves the capacity to accommodate external RNA molecules as templates for aminoacyl-T RNA binding.

**Figure 15:**
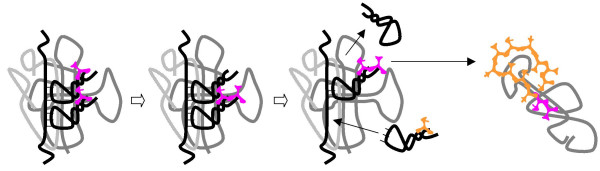
Origin of the translation system and the genetic code in the RNA World. Step 11: The **R**_**L**_**R**_**S **_complex (the protoribosome) evolves the mRNA translocation mechanism. A primitive version of translation evolves.

9. The physical separation of the binding and catalytic functions of **R **– with the binding/recognition of aminoacyl-**T **RNAs now relegated to **R**_**S **_and the transpeptidation reaction remaining the function of **R**_**L **_– provides for the separate evolution and elaboration of the respective activities (a situation that could be viewed as a distinct type of subfunctionalization). In particular, new forms of **R**_**S **_would evolve by duplication and diversification, increasing the repertoire of peptides produced by the **R**_**S**_**R**_**L **_complex (Fig. 13). At this and the next step, selection for the most beneficial peptides would "freeze" the correspondence between the anticodon of the RNA **T **and the identity of the associated amino acid under FAM.

10. As the next step, we envision the physical separation of the template strand **M **from **R**_**S**_, resulting in further disentanglement of coding and catalysis (Fig. 14). At this point, the strand **M **is practically free from evolutionary constraints associated with the binding and catalytic activities involved in the primitive translation because all of these functions are provided by physically distinct RNA molecules, **R**_**L**_, **R**_**S**_, and the proto-tRNAs. The only requirement for **M **is to adopt a semi-extended conformation to accommodate the codon-anticodon base-pairing that is required for the binding of an aminoacyl-**T **RNA. The selective benefits of such separation are obvious: transient association of **R**_**S**_**R**_**L **_(which, at this point, we will call the proto-ribosome for the first time) with different oligo/polynucleotides present in the compartment would lead to the production of an increasing variety of peptides, thus enhancing the catalytic potential of the ensemble. Furthermore, this step would enable the selection for improved replication potential (e.g., high-affinity replicase recognition sites) of those species of **M **that encode useful peptides, leading to enrichment of these RNA species in the compartment. Thus, an RNA-peptide Eigen- Schuster hypercycle [6], a distinct version of the Darwin-Eigen cycle, would be effectively established within the selfish cooperative.

11. Conceivably, the release of a discharged (proto)tRNA from **R**_**S**_**R**_**L **_upon trans-peptidation triggered the trinucleotide shift, the crucial movement performed by modern ribosomes [19,124] and allowing for the synthesis of longer peptides, i.e., the first proteins (Fig. 15). This is when the Protein Breakthrough takes place.

The evolutionary path from the breakthrough stage outlined above to the modern-type translation system was, largely, a story of takeover of the primordial ribozyme functions by evolving proteins. Proteins have an incomparably greater potential for evolution of diverse binding and catalytic capacities than peptides or RNA and, accordingly, they soon began to gradually supplant the ribozymes. Given the greater chemical versatility and efficiency of proteins as catalysts, each such displacement is irreversible, as insightfully stressed by Penny [8].

The rest, as they say, is history.

## Discussion and conclusion

### The status of the model: incentives and constraints

The scenarios for the origin of the translation system and the genetic code outlined here are both sketchy and highly speculative. Why, then, bother building such conceptual, qualitative models at all? The justification for this kind of theorizing can be succinctly put in the short phrase: we have to get from ***there ***to ***here***. ***There ***being the early, cooling earth with no complex organic molecules, and ***here ***being a minimally complex genetic system with modern-type translation, transcription, and replication machineries, a system that would be subject to biological evolution much like modern organisms. The replication and transcription problems are, at least, logically relatively straightforward, even if hard from the chemical point of view, inasmuch as no new principles, beyond base complementarity, and enzymatic catalysis need to be invented. Thus, plausible, even if conflicting, accounts of the emergence of these systems have been derived from comparative-genomic data and evolutionary reasoning [70,140-144]. There is, however, a crucial snag about these models: they all rely on a pre-existing translation system. And the origin of the translation system is far from being a trivial matter. The main difficulty is not even its complexity per se but the necessity to invent a new principle, that of the genetic code, the correspondence between the *a priori *unconnected sequences of nucleotides and amino acids. It might not be much of an exaggeration to note that, at least, at first glance, the origin of the translation system evokes the scary specter of irreducible complexity.

Thus, our main incentive with the present analysis was to deconstruct the formidable problem of the emergence of translation into a series of plausible and manageable steps, in accordance with the Continuity Principle. We believe that, in doing so, we achieved a somewhat greater level of detail and coherence than any of the previous models we are aware of. Importantly, in constructing this model, we were both constrained and driven by: i) comparative-genomic data, ii) experimental data on amino-acid-codon recognition, iii) experimental data on the diverse catalytic activities of ribozymes.

Comparative-genomic analysis indicates that an elaborate translation system, comparable to the modern one in terms of fidelity and efficiency, has evolved within the RNA world. Indeed, extensive diversification of many protein folds occurred before the advent of some of the essential components of the modern translation system, such as aaRS and translation factors. Before the emergence of these dedicated proteins, the translation system must have been a machine comprised primarily, if not exclusively, of RNA. The only conceivable alternative, that the primordial translation system employed a different, currently, extinct complement of essential protein factors, inevitably leads to infinite regression. Thus, it seems to be a virtually inevitable conclusion that the ancient, RNA-only translation system was comparable in efficiency to the modern one. This might seem paradoxical and even not credible at a superficial glance. However, a quick reflection suggests that: i) the skeleton of the modern translation system actually consists of RNA, with the proteins being elaborations, however numerous and important, and ii) logically, it hardly could have been otherwise: indeed, in order to switch to a new type of constituents (proteins), biological systems needed the means to produce them accurately. It is conceivable and, indeed, likely that peptides produced by the first, RNA-based proto-translation systems provided positive feedback leading to hypercycle formation (Figs. 4, 6). However, this primitive version of translation must have been quite sloppy and hardly could master production of anything beyond relatively short peptides. Evolution of the (nearly) complete set of tRNAs was a pre-requisite for achieving the fidelity required to kick off protein evolution in earnest.

In our description of the model, the alternative scenarios based on CRM, ARM, and FAM are considered on equal footing. As discussed above, the currently available data are too ambiguous to conclude which of these models for the origin of coding is most likely. However, it should be noted that, important as they are in terms of the actual physico-chemical underpinning of the code, the differences between CRM, ARM, and FAM do not translate into major modifications of the evolutionary scenario. Indeed, the central principles remains the same, i.e., specific recognition of amino acids by proto-tRNAs such that an amino acid is paired with the cognate anticodon with sufficient reliability.

### Lasting principles and ephemeral details

The models presented here were deliberately constructed at the level of considerable detail -at the risk of getting many, perhaps, most aspects wrong – in order to provide a proof of principle, i.e., to illustrate a plausible sequence of selectively advantageous steps along the path from the RNA world to the modern-type translation system. This being said, there seem to be several underlying principles that are likely to stand regardless of further developments. We briefly recapitulate these:

1. Evolution having no foresight, selection for translation *per se *is not feasible.

Translation must have evolved as a by-product of selection for some other function, i.e., via the exaptation route.

2. Given that the essence of translation is the intimate link between RNA and proteins, it seems most likely that, in some form, this connection existed from the very beginning of the evolutionary path from the RNA World to translation. Thus, the proposed starting point, i.e., stimulation of ribozymes by amino acids and peptides seems to be a strong, almost, logically required, candidate for this role (see also [145]).

3. Synthesis of peptides directly on an RNA template is stereochemically unfeasible. Hence adaptors must have been part of the primordial translation system from the start. Accordingly, from the very onset of translation, adaptors have been key to the establishment of the genetic code. These ancestral adaptors, although, in all likelihood, smaller and simpler than modern tRNAs, must have been endowed with catalytic capacities lacking in the latter, i.e., they would have to catalyze specific self-aminoacylation with the cognate amino acids.

4. The primordial translation system was dominated by RNA although peptides might facilitate its functioning. However, the fidelity of this primordial, (nearly) RNA-only translation system must have been comparable to that of modern translation systems, considering that extensive protein evolution took place prior to the diversification of the proteins that are essential for the modern translation.

### Problems and testability

The current scenario for the evolution of translation in the RNA World faces formidable difficulties because, although the ribozyme catalysis of the elementary reactions required for translation has been demonstrated experimentally (Table 1), the required complex RNA-mediated functions have not. The crux of the problem seems to lie in the postulated catalytic adaptors that would have to possess a notable spectrum of capabilities including, in addition to the apparently feasible specific recognition of amino acids and self-aminoacylation, the ordered binding to the progenitor of the large subunit (**R**_**L**_), and at a subsequent stage, recognition of a specific region in the progenitor of the small subunit (**R**_**S**_). With regard to **R**_**L **_and **R**_**S **_themselves, ribozyme stimulation by amino acids and peptides has been demonstrated but, beyond that, the postulated properties of these molecules remain hypothetical. It seems that a focused experimental effort aimed at the construction/selection of ribozymes with the properties of the postulated **T **RNAs, in particular, their postulated interaction with other, more complex ribozymes, could provide crucial evidence in support of this or a similar scenario for the evolution of translation.

Although the individual ribozyme-catalyzed reactions involved in the postulated scheme are feasible, the succession of multiple evolutionary steps that appear to be required for the emergence of translation might be legitimately viewed as far fetched, particularly, considering the inevitably inefficient ribozyme-mediated replication that must have been prevalent in the RNA World. Be as it may, this is, at present, our best effort to develop a conceptual model for the origin of translation. Elsewhere, one of us (EVK) examines a radical alternative [17].

## Reviewers' comments

Reviewer 1: Rob Knight (University of Colorado)

In this intriguing manuscript, Wolf & Koonin combine comparative genomics with Eigen's (1978) concept of the error threshold to provide a new, comprehensive model for the origins of translation. Specifically, they build on Szathmary's (1993) model of amino acids as coenzymes in an RNA metabolism as a starting point for the genetic code. As pointed out by Knight & Landweber (2000), there are three pathways to a protein-based genetic code from the RNA world that preserves continuity of features of the genetic code: the RNAs that bind directly could have played the roles of tRNAs, mRNAs, or aminoacyl-tRNA synthetases. Wolf & Koonin favor a model along the lines of the latter role, suggesting that cofactor-enhanced catalysis, and then nonribosomal synthesis of short peptides, were the original driving force for RNA-catalyzed translation. They present an intriguing new overall model of the evolution of the translation system, and highlight aspects of this model that could be tested in the laboratory. The main weakness of the manuscript in its current form is its endorsement of the frozen accident model (FAM) of the genetic code's evolution without the presentation of alternative explanations of the evidence in favor of the optimality of the genetic code relative to random codes, and the coding triplet/binding site associations that have been observed through SELEX and in the Group I intron. However, as the authors themselves point out, the resurrection of the frozen accident model is not an important feature of their overall model for the emergence of translation, and this discussion could be omitted without diminishing the manuscript's contribution.

The manuscript presents some interesting ideas that I have not seen elsewhere and that appear to shed substantial new light on the difficult problem of the origin of translation.

For example, the discussion on p. 13 that shows that the domains in the aaRS are highly derived relative to domains in other proteins is extremely interesting, because we might have expected the aaRS to be among the earliest proteins. If they are not, the likelihood that they displaced some other system for coded translation increases dramatically (Theobald & Wuttke's 2005 study of OB-fold superfamily relationships also supports this idea). One point that should be specifically noted in this context is that not only do these relationships imply that the aaRS are relatively late arrivals, but also that coded translation must have predated the aaRS so that the sequence information that allows us to determine the phylogenetic relationships among these folds could be transmitted to the present. In other words, if comparable folds were once produced by a different synthesis mechanism, either we would need either a system of reverse translation to copy the sequence information into nucleic acids, or all of the proteins produced by that mechanism would have been lost when coded translation took over.

Similarly, the discussion on pp. 33–39 of a plausible scenario for the evolution of the modern translation system seems plausible and is more detailed than most such scenarios to be found in the literature.

A couple of areas of the manuscript could potentially be supported by drawing on additional literature. For example, on p. 8, Dennett has an excellent discussion in "Darwin's Dangerous Idea" (Simon & Schuster, 1995) of the production of apparently irreducibly complex phenomena through simplification of an even more complex system, e.g. building an arch by taking away stones from a pile of rubble. The complexity of the system of peptide- specific synthetases that would be required for the model proposed here might make this an appropriate metaphor. Similarly, Yarus's (2001) article "On translation by RNAs alone", and Yarus & Welch's (2000) article "Peptidyl transferase: ancient and exiguous" contain some thoughts that would be relevant here and later in the manuscript.

**Author response: ***Dennett's metaphor of the Roman arch is, indeed, excellent and might be relevant, even if not directly, because, here, we are talking more of stepwise displacement than selective elimination, and do not really postulate an initial state that was more complex than the final one. In any case, one of the strengths of the Biology Direct model is that the review is published, so the reader can read about this metaphor here. Ditto for the reviews by Yarus: the reader now knows of them and may turn to them if desirable (other work from Yarus' laboratory is cited extensively)*.

The discussion of ribozymes on p. 18 could possibly benefit from a discussion of riboswitches and their implications for control mechanisms in the cell, and/or for the other roles or RNA that suggest the RNA World (use in cofactors, role in nucleotide metabolism, use of RNA as a primer in DNA synthesis, etc.) However, the manuscript is fairly long as it is, and most of these points have been raised many times in the cited literature already.

**Author response: ***Yes, the paper is fairly long, and we believe that riboswtiches are of no direct relevance*.

Finally, some of the specific contentions could benefit from more elaboration. For example, on pp. 11–12, we find the statement:

"Put another way, the conservation of the core of the translation machinery is the strongest available evidence that some form of LUCA actually existed (it is, in principle, conceivable that life started off as a multitude of distinct forms but a single variant of the translation system subsequently took over as a result of a sweeping horizontal gene transfer; however, this is a decidedly non- parsimonious scenario)."

Given that the present manuscript already proposes the evolution of an entire suite of RNA-based aminoacyl-tRNA synthetases that no longer exist, and given that some authors such as Carl Woese propose that the division of life into distinct phylogenetic lineages was a relatively late event (e.g. Woese 2002), it is unclear why horizontal gene transfer should be dismissed in this context.

**Author response: ***Upon more careful consideration (also considering Mushegian's comments below), we have deleted this whole claim. Suffice it to say, in this context, that the conservation of the translation machinery is evidence of ***some ***form of LUCA*.

Similarly, on p. 20, the authors seem to be strongly in favor of the hydrothermal vent scenario for the origin of life. A few words of caution to the effect that this is one of many hypotheses for life's origin, and that data are still far from conclusive, might be in order.

**Author response: ***we have included a few words to that effect but also cite new references that, we believe, add credibility to the hydrothermal vent scenario (refs. 75, 76)*.

The discussion of the current evidence relating to the hypothesis that the genetic code arose through direct interactions between RNA and amino acids on p. 23 is good, but on p. 41 we read that "these affinities are weak, only manifest as a statistical trend, and worst of all, are seen, mostly, for chemically complex amino acids like arginine or histidine, rather than simple ones, such as glycine or alanine, that would be readily produced abiogenically." This statement requires some elaboration. Many of the potentially prebiotic amino acids, such as glycine, are difficult to evaluate with the affinity chromatography paradigm for technical reasons. It is possible that other methodologies, such as the allosteric selections pioneered by Tang & Breaker (1997), will allow us to see interactions in these cases, but for now absence of evidence should not be taken as evidence of absence. It is also far from certain that the biosynthesis of complex amino acids such as arginine would have been beyond the capabilities of RNA World organisms, so the primordial genetic code need not have been confined to simple amino acids. Second, the physical interactions involved are often far from weak: some amino acid aptamers, such as the best of Famulok's (1996) arginine aptamers, have sub-micromolar dissociation constants. It is true that the inconsistency between codon and anticodon modes of recognition remains to be resolved, but I do not agree with the assertion that "objectively, we should accept FAM as the most likely model for the emergence and evolution of translation". To accept FAM given what we know now about the optimality of the genetic code relative to random genetic codes, and the relationships between amino acid binding sites and cognate triplets, requires an alternative explanation for the strong statistical evidence that supports these hypotheses. In the absence of such an alternative explanation for why we see these patterns, which would be extremely unlikely under the FAM, I would recommend that the discussion be confined to pointing out where these processes would most likely be able to act in the model (for example, everyone agrees that direct interactions between coding triplets and amino acids are not relevant to the modern genetic code). It is possible that FAM is not an optimal description of what is actually meant in the discussion in the text – really, the claim seems to be that there is no necessary relationship between triplets of RNA and amino acids, rather than that there is in fact no pattern. However, in my opinion, the discussion of FAM vs. ARM vs. CRM as presented is likely to be a distraction from the overall value of the new ideas presented in the manuscript.

**Author response: ***We cannot agree that this description is a distraction; we think it is part and parcel of the paper, even if the choice between ARM, CRM, and FAM has a limited effect on the actual model considered here. However, this discussion has been shortened and modified to make it more neutral with regard to the choice between the model of amino acid- T RNA recognition. The statement regarding weak interactions between amino acids and aptamers has been dropped along with the over-assertive statement regarding FAM as "*the most likely model"*. It seems like in the text we clearly explain what we mean by FAM – indeed, it is about a lack of any direct connection between amino acids and cognate triplet. Also, we consider the amended version of FAM where subsequent adaptation of the code is deemed likely*.

Finally, the description of experimental tests on p44 could benefit from more detail. Which properties of the postulated T RNAs are in doubt, and which steps would, if experimentally confirmed, best support the model? More specific guidance might increase the probability that supporting laboratory work would be carried out.

**Author response: ***A brief discussion has been added*.

Reviewer 2: Doron Lancet (Weizmann Institute of Science)

This reviewer made no comments.

Reviewer 3: Alexander Mankin, University of Illinois at Chicago (nominated by Arcady Mushegian)

It is a fairly straightforward task to evaluate an experimental paper driven by the data. It is a much more fuzzy assignment to evaluate a theoretical paper discussing a possible evolutionary scenario of the origin of protein synthesis. It is very tempting to buy into all of the authors' arguments. It is equally tempting to criticize them all.

The main postulate of Wolf and Koonin is that they are trying to build a model based on the Continuity Principle. In lay language, this means they are trying to put little solid rocks into the vast swamp that separates the evolutionary island of the RNA World, where most of the biochemical reactions are catalyzed by ribozymes, from the island of the modern nucleic acid-protein world, where biochemistry is carried out primarily by protein enzymes whilst nucleic acids are involved mostly in storage and expression of genetic information. Trying to bridge this gap, the authors envision the intermediate steps on the evolutionary path to the genetic code and coded protein synthesis, where innovations that arose at each of the steps could be selected for. In this approach, Wolf and Koonin strive to allow for the fewest number of evolutionary gaps that would require a significant leap rather than a small jump. Not that this is a new approach – most of the previous attempts to delineate the origin of protein synthesis were based on a generally similar idea. However, in the prior works, it was probably more of an intuitive attempt to build a plausible scenario than a formulated goal as in the essay of Wolf and Koonin.

The question is how closely those rocks of Wolf and Koonin are spaced and how solid they are. Some of them appear to be nicely positioned and are fairly solid, whereas the others, in my view, are either shaky or missing.

It seems to be a very reasonable idea that some of the RNA World ribozymes could benefit from a bound amino acid cofactor or even cofactors. It appears to be a much more far-fetched speculation that two or even more of these cofactors would bind in such close proximity of each other that the formation of a peptide bond between them would be possible and beneficial. Furthermore, it is not entirely clear from where a hypothetical peptide ligase would derive the energy that is required for peptide bond formation. In the modern ribosome, the energy that powers peptide bond formation is conserved in the high-energy ester bond that links the C-terminal amino acid of a nascent peptide to tRNA. The energy of this ester bond is derived from ATP consumed by an aminoacyl-tRNA synthetase – a source hardly available in the RNA world.

**Author response: ***Yes, the issue of the energy source is important. One would have to propose that one of the substrates of the primordial peptide ligase was an activated amino acid, perhaps, even an aminoacyl adenylate. In the RNA world, such derivatives would have to be produced by other ribozymes, and ribozymes with such an activity, indeed, have been described (see *Table 1). *Alternatively, the original ribozyme R might have been an ATPase such that the emerging peptide ligase would couple ATP hydrolysis with peptide synthesis. The text was amended to address these issues*.

Though the proposed route that leads to the origin of the original peptide ligase/aminoacyl polymerase is questionable, the resulting entity – a ribozyme capable of polymerizing amino acids into peptides in an unprogrammed fashion – seems highly plausible. As early experiments of Monro have shown, the large ribosomal subunit of the modern ribosome, a ribozyme in its own right, is still capable of carrying out such a reaction if provided with properly activated amino acids. So, if one is to accept Wolf and Koonin's idea of a peptide ligase derived from a ribozyme that is able to connect its amino acid cofactors into a single peptide, then the next few steps in their scenario are rather convincing. The use of the resulting peptides by other ribozymes, a subfunctionalization of the original peptide-ligating ribozyme into a specialized peptide ligase or amino acid polymerase, and the general benefit of having such a peptide ligase ribozyme in the assembly of selfish cooperatives appear to pave a rather smooth path for the ancestor of the large ribosomal subunit.

Having 'prepared' the key catalyst of protein synthesis, Wolf and Koonin then address the problem of a tRNA adaptor. An elegant idea they propose to justify the evolutionary necessity for establishing a link between pre-tRNAs and amino acids is that this would limit the diffusibility of a small amino acids and would help to increase their local concentration. Given that ribozymes with tRNA aminoacylating activities have been identified in SELEX experiments, it is easy to imagine that ribozymes with similar activities could have been selected through natural evolution in the RNA World. When considering the correspondence between the tRNA anticodon and the amino acid, Wolf and Koonin chose to not take sides in the discussion of whether the origin of the genetic code is based on a chemical complementarity between an amino acid and a codon or anticodon or is a result of a frozen evolutionary accident. Though the all-inclusive approach inevitably makes the description of this step somewhat fuzzy, any of several scenarios mentioned in this section are pleasantly consistent and provide good food for thought.

The next step is equally convincing: the invention of aminoacyl-tRNA organically leads to its use by the prototype peptide ligating/aminoacyl polymerizing ribozyme and thus completes the route to the large ribosomal subunit ancestor.

The origin of the coded protein synthesis is based on availability of three main players: the adaptor aminoacyl-RNA molecules with a strict amino acid-anticodon correlation, an enzyme that can polymerize the activated amino acids (the large ribosomal subunit precursor), and a precursor of the small ribosomal subunit, a "reading head" that selects the adaptor aminoacyl-RNA according to the input genetic text. Wolf and Koonin derive the origin of the ancestor of the small ribosomal subunit not from a pre-existing ribozyme but from a segment of the large subunit precursor. In this 'Adam's rib' scenario, an accessory RNA subunit R_S _evolves as a tool to enhance binding and positioning of aminoacyl-tRNA on the catalytic subunit, then acquires the "burden of specific recognition," and later on, one of its own parts assumes the role of a diffusible template. I am not sure whether this, rather sketchy scenario, satisfies the acclaimed Continuity Principle. Furthermore, it is poorly supported by the fact that the modern large ribosomal subunit can rather efficiently catalyze peptide bond formation using tRNA substrates even in the absence of the small subunit (Wohlgemuth, Beringer, Rodnina, (2006) EMBO Rep., 7, 699–703). From the point of view of this reviewer, it is more reasonable to root the origin of the small subunit in one of the pre-existing ribozymes that could operate with RNA templates. The extant activities of the modern small ribosomal subunit, including its interaction with an RNA template (mRNA) and ability to assemble on it the complementary sequences of the tRNA anticodons, bear the features expected from the ancestral RNA replicase/RNA ligase. Such a ribozyme could be viewed as an ancestor of the ribosome decoding center. The suspected ability of the modern 30 S subunit to cleave mRNA during ribosome stalling or under the influence of specific protein factors argues that the putative ancient catalytic center capable of breaking (and thus forming) phosphodiester bonds may still exist in the ribosome.

**Author response: ***The possibility that the small subunit of the ribosome evolved from an RNA replicase/triplicase is an interesting one, and we have considered a version of it when working on the current model. This could directly connect the model discussed here with the triplicase model of Pool-Jeffares-Penny. However...direct evidence is missing, so we decided to avoid "overfitting" the model. Let the reader learn about this idea from Mankin's comment. However, it is completely unclear to us why the work of Wohlgemuth et al. is construed as evidence against the model presented in the paper. We believe that, on the contrary, it is readily compatible with this model, and we cite it in the revision*.

In conclusion, the essay of Wolf and Koonin is an interesting and highly stimulating work. Inadvertently, my review sounds more critical than was intended. The reason is simple: the ideas we disagree with are more interesting for us than the points we easily accept. The majority of the points in the paper are of this latter category; the points my comments mostly focus on are of the former.

Other points of critique and comments:

1. The discussion of the model *per se *starts on p. 28. It seems that an almost 30-page introduction is excessive and often repetitive. The work would strongly benefit if the first 28 pages were expressed more succinctly, possibly as bulleted points in 2 pages.

**Author response: ***We appreciate the virtues of brevity but this paper was conceived as a specific model for the origin of translation placed against the critically examined background of the relevant general evolutionary principles and previous research in the area. We feel that it has to stay that way*.

Reviewer #4: Arcady Mushegian

The most significant contribution of this study is in decomposing the tantalizingly complex problem of the origin of genetic code, translation, and RNA replication into a series of proposed small evolutionary transitions, each associated with its own contribution to the fitness of the genetic system that experiences these transitions. I whole-heartedly recommend this manuscript for publication and expect that this series of transitions will be further scrutinized, perhaps along the lines of necessity and sufficiency.

My only scientific complain is about the half-haphazard conclusion that the frozen-accident model of adaptor recognition by amino acids is the most likely one. It might be, or it might be not: the fact that current direct experiments fail to establish specific recognition of cognate (anti)codons for evolutionarily more primitive amino acids does not make a "frozen accident" mechanistically attractive. Moreover, if, for example, primitive nucleobases were abiotically derivatized (see the work from S.Benner's lab that seems to point in this direction), then the experiments with the present-day codons or anticodons are not even answering the right question. The authors should mention that work or at least stay even more agnostic about the recognition model.

**Author response: ***we infused considerable extra agnosticism, also, in response to Knight's comments (see above)*.

Other, minor, comments:

"The Continuity Principle" has connections with Anton Dorn's change-of-function principle (Ursprung der Wirbeltiere und das Prinzip des Funktionswechsels, Leipzig, 1875) – perhaps this is worth acknowledging.

**Author response: ***In truth, the principle really goes back to Darwin, the rest are reformulations and explanations. We jump to a modern version immediately, leaving Dorn out*.

As discussed by the authors, should Darwin-Eigen cycle be renamed Darvin-Eigen-Lynch-Conery cycle?

**Author response: ***If one wants to be really fair, then, maybe, Darwin-Eigen-Penny-Lynch-Conery -(Wolf-Koonin)? For the time being, we are sticking with the original name, after Penny*.

The study is well-written, but perhaps it can be edited a bit more. For example, the notion that "evolution has no foresight", however important, is seen at least five times, including two times within one bulleted list on pg 29.

## Individual authors' contributions

YIW incepted the study, developed the initial version of the model, and wrote the first draft of the model description; YIW and EVK jointly finalized the model; EVK wrote the manuscript. All authors have read and approved the final version of the manuscript.

## References

[B1] Darwin C (1859). On the Origin of Species by Means of Natural Selection or, The Preservation of Races in the Struggle for Life..

[B2] Crick FH, Brenner S, Klug A, Pieczenik G (1976). A speculation on the origin of protein synthesis. Orig Life.

[B3] Eigen M (1971). Selforganization of matter and the evolution of biological macromolecules. Naturwissenschaften.

[B4] Orgel LE (1963). The maintenance of the accuracy of protein synthesis and its relevance to ageing. Proc Natl Acad Sci U S A.

[B5] Jeffares DC, Poole AM, Penny D (1998). Relics from the RNA world. J Mol Evol.

[B6] Eigen M, Schuster P (1977). The hypercycle. A principle of natural self-organization. Part A: Emergence of the hypercycle. Naturwissenschaften.

[B7] Smith JM (1979). Hypercycles and the origin of life. Nature.

[B8] Penny D (2005). An Interpretive Review of the Origin of Life Research. Philos Biol.

[B9] Lynch M, Conery JS (2003). The origins of genome complexity. Science.

[B10] Domingo E, Biebricher CK, Eigen M, Holland JJ (2002). Quasispecies and RNA Virus Evolution: Principles and Consequences.

[B11] Gould SJ (1997). The exaptive excellence of spandrels as a term and prototype. Proc Natl Acad Sci U S A.

[B12] Gould SJ, Lewontin RC (1979). The spandrels of San Marco and the Panglossian paradigm: a critique of the adaptationist programme. Proc R Soc Lond B Biol Sci.

[B13] Koonin EV, Aravind L, Kondrashov AS (2000). The impact of comparative genomics on our understanding of evolution. Cell.

[B14] Kimura M (1983). The Neutral Theory of Molecular Evolution.

[B15] Crick FH (1958). On protein synthesis. Symp Soc Exp Biol.

[B16] Crick F (1970). Central dogma of molecular biology. Nature.

[B17] Koonin EV (2007). The cosmological model of eternal inflation and the  transition from chance to biological evolution in the history of life.. Biol Direct.

[B18] Spirin AS (1999). Ribosomes.

[B19] Spirin AS (2004). The ribosome as an RNA-based molecular machine. RNA Biology.

[B20] Selmer M, Dunham CM, Murphy FV, Weixlbaumer A, Petry S, Kelley AC, Weir JR, Ramakrishnan V (2006). Structure of the 70S ribosome complexed with mRNA and tRNA. Science.

[B21] Ramakrishnan V (2002). Ribosome structure and the mechanism of translation. Cell.

[B22] Moore PB, Steitz TA (2005). The ribosome revealed. Trends Biochem Sci.

[B23] Koonin EV (2003). Comparative genomics, minimal gene-sets and the last universal common ancestor. Nat Rev Microbiol.

[B24] Anantharaman V, Koonin EV, Aravind L (2002). Comparative genomics and evolution of proteins involved in RNA metabolism. Nucleic Acids Res.

[B25] Wolf YI, Aravind L, Grishin NV, Koonin EV (1999). Evolution of aminoacyl-tRNA synthetases--analysis of unique domain architectures and phylogenetic trees reveals a complex history of horizontal gene transfer events. Genome Res.

[B26] Woese CR, Olsen GJ, Ibba M, Soll D (2000). Aminoacyl-tRNA synthetases, the genetic code, and the evolutionary process. Microbiol Mol Biol Rev.

[B27] Aravind L, Anantharaman V, Koonin EV (2002). Monophyly of class I aminoacyl tRNA synthetase, USPA, ETFP, photolyase, and PP-ATPase nucleotide-binding domains: implications for protein evolution in the RNA. Proteins.

[B28] Aravind L, Mazumder R, Vasudevan S, Koonin EV (2002). Trends in protein evolution inferred from sequence and structure analysis. Curr Opin Struct Biol.

[B29] Artymiuk PJ, Poirrette AR, Rice DW, Willett P (1996). Biotin carboxylase comes into the fold. Nat Struct Biol.

[B30] Leipe DD, Wolf YI, Koonin EV, Aravind L (2002). Classification and evolution of P-loop GTPases and related ATPases. J Mol Biol.

[B31] Noller HF, Gesteland RF, Cech TR, Atkins JF (2006). Evolution of ribosomes and translation from an RNA world. The RNA World.

[B32] Fitch WM, Upper K (1987). The phylogeny of tRNA sequences provides evidence for ambiguity reduction in the origin of the genetic code. Cold Spring Harb Symp Quant Biol.

[B33] Eigen M, Lindemann BF, Tietze M, Winkler-Oswatitsch R, Dress A, von Haeseler A (1989). How old is the genetic code? Statistical geometry of tRNA provides an answer. Science.

[B34] Eigen M, Winkler-Oswatitsch R (1981). Transfer-RNA: the early adaptor. Naturwissenschaften.

[B35] Woese CR (1967). The Genetic Code.

[B36] Crick FH (1968). The origin of the genetic code. J Mol Biol.

[B37] Orgel LE (1968). Evolution of the genetic apparatus. J Mol Biol.

[B38] Kruger K, Grabowski PJ, Zaug AJ, Sands J, Gottschling DE, Cech TR (1982). Self-splicing RNA: autoexcision and autocyclization of the ribosomal RNA intervening sequence of Tetrahymena. Cell.

[B39] Guerrier-Takada C, Gardiner K, Marsh T, Pace N, Altman S (1983). The RNA moiety of ribonuclease P is the catalytic subunit of the enzyme. Cell.

[B40] Fedor MJ, Williamson JR (2005). The catalytic diversity of RNAs. Nat Rev Mol Cell Biol.

[B41] Doudna JA, Lorsch JR (2005). Ribozyme catalysis: not different, just worse. Nat Struct Mol Biol.

[B42] Lilley DM (2005). Structure, folding and mechanisms of ribozymes. Curr Opin Struct Biol.

[B43] Muller UF (2006). Re-creating an RNA world. Cell Mol Life Sci.

[B44] Gilbert W (1986). The RNA World. Nature.

[B45] Joyce GF (2002). The antiquity of RNA-based evolution. Nature.

[B46] Orgel LE (2004). Prebiotic chemistry and the origin of the RNA world. Crit Rev Biochem Mol Biol.

[B47] Orgel LE (2003). Some consequences of the RNA world hypothesis. Orig Life Evol Biosph.

[B48] Gesteland RF, Cech TR, Atkins JF, Cold Spring Harbor (2006). The RNA World.

[B49] Joyce GF (2004). Directed evolution of nucleic acid enzymes. Annu Rev Biochem.

[B50] Beaudry AA, Joyce GF (1992). Directed evolution of an RNA enzyme. Science.

[B51] Illangasekare M, Yarus M (1999). Specific, rapid synthesis of Phe-RNA by RNA. Proc Natl Acad Sci U S A.

[B52] Johnston WK, Unrau PJ, Lawrence MS, Glasner ME, Bartel DP (2001). RNA-catalyzed RNA polymerization: accurate and general RNA-templated primer extension. Science.

[B53] Lawrence MS, Bartel DP (2005). New ligase-derived RNA polymerase ribozymes. Rna.

[B54] Lawrence MS, Bartel DP (2003). Processivity of ribozyme-catalyzed RNA polymerization. Biochemistry.

[B55] McGinness KE, Wright MC, Joyce GF (2002). Continuous in vitro evolution of a ribozyme that catalyzes three successive nucleotidyl addition reactions. Chem Biol.

[B56] McGinness KE, Joyce GF (2003). In search of an RNA replicase ribozyme. Chem Biol.

[B57] Doudna JA, Cech TR (2002). The chemical repertoire of natural ribozymes. Nature.

[B58] Brosius J (2005). Echoes from the past--are we still in an RNP world?. Cytogenet Genome Res.

[B59] Steitz TA, Moore PB (2003). RNA, the first macromolecular catalyst: the ribosome is a ribozyme. Trends Biochem Sci.

[B60] Moore PB, Steitz TA (2002). The involvement of RNA in ribosome function. Nature.

[B61] Polacek N, Mankin AS (2005). The ribosomal peptidyl transferase center: structure, function, evolution, inhibition. Crit Rev Biochem Mol Biol.

[B62] Kirsebom LA (2002). RNase P RNA-mediated catalysis. Biochem Soc Trans.

[B63] Altman S, Baer MF, Bartkiewicz M, Gold H, Guerrier-Takada C, Kirsebom LA, Lumelsky N, Peck K (1989). Catalysis by the RNA subunit of RNase P--a minireview. Gene.

[B64] Woodson SA (2005). Structure and assembly of group I introns. Curr Opin Struct Biol.

[B65] Haugen P, Simon DM, Bhattacharya D (2005). The natural history of group I introns. Trends Genet.

[B66] Robart AR, Zimmerly S (2005). Group II intron retroelements: function and diversity. Cytogenet Genome Res.

[B67] Dai L, Toor N, Olson R, Keeping A, Zimmerly S (2003). Database for mobile group II introns. Nucleic Acids Res.

[B68] Lambowitz AM, Zimmerly S (2004). Mobile group II introns. Annu Rev Genet.

[B69] Lehmann K, Schmidt U (2003). Group II introns: structure and catalytic versatility of large natural ribozymes. Crit Rev Biochem Mol Biol.

[B70] Koonin EV, Senkevich TG, Dolja VV (2006). The ancient virus world and evolution of cells. Biol Direct.

[B71] Flores R, Delgado S, Gas ME, Carbonell A, Molina D, Gago S, De la Pena M (2004). Viroids: the minimal non-coding RNAs with autonomous replication. FEBS Lett.

[B72] Russell MJ, Hall AJ (1997). The emergence of life from iron monosulphide bubbles at a submarine hydrothermal redox and pH front. J Geol Soc London.

[B73] Martin W, Russell MJ (2003). On the origins of cells: a hypothesis for the evolutionary transitions from abiotic geochemistry to chemoautotrophic prokaryotes, and from prokaryotes to nucleated cells. Philos Trans R Soc Lond B Biol Sci.

[B74] Koonin EV, Martin W (2005). On the origin of genomes and cells within inorganic compartments. Trends Genet.

[B75] Baaske P, Weinert F, Duhr S, Lemke KH, Russell MJ, Braun D (2007). Extreme accumulation of nucleotides in simulated hydrothermal pore systems. Proc Natl Acad Sci U S A.

[B76] Koonin EV (2007). An RNA-making reactor for the origin of life. Proc Natl Acad Sci U S A.

[B77] Kun A, Santos M, Szathmary E (2005). Real ribozymes suggest a relaxed error threshold. Nat Genet.

[B78] Muller UF, Bartel DP (2003). Substrate 2'-hydroxyl groups required for ribozyme-catalyzed polymerization. Chem Biol.

[B79] Poole AM (2006). Getting from an RNA world to modern cells just got a little easier. Bioessays.

[B80] Woese CR (1962). Nature of the biological code. Nature.

[B81] Woese CR (1965). On the evolution of the genetic code. Proc Natl Acad Sci U S A.

[B82] Woese CR (1965). Order in the genetic code. Proc Natl Acad Sci U S A.

[B83] Sonneborn TM, Bryson, V., Vogel HJ (1965). Evolution of the genetic code. Evolving Genes and Proteins.

[B84] Knight RD, Freeland SJ, Landweber LF (1999). Selection, history and chemistry: the three faces of the genetic code. Trends Biochem Sci.

[B85] Di Giulio M (2005). The origin of the genetic code: theories and their relationships, a review. Biosystems.

[B86] Ycas M (1999). Codons and hypercycles. Orig Life Evol Biosph.

[B87] Maeshiro T, Kimura M (1998). The role of robustness and changeability on the origin and evolution of genetic codes. Proc Natl Acad Sci U S A.

[B88] Lehmann J (2000). Physico-chemical constraints connected with the coding properties of the genetic system. J Theor Biol.

[B89] Freeland SJ, Hurst LD (1998). The genetic code is one in a million. J Mol Evol.

[B90] Freeland SJ, Knight RD, Landweber LF, Hurst LD (2000). Early fixation of an optimal genetic code. Mol Biol Evol.

[B91] Haig D, Hurst LD (1991). A quantitative measure of error minimization in the genetic code. J Mol Evol.

[B92] Haig D, Hurst LD (1999). A quantitative measure of error minimization in the genetic code. J Mol Evol.

[B93] Freeland SJ, Wu T, Keulmann N (2003). The case for an error minimizing standard genetic code. Orig Life Evol Biosph.

[B94] Woese CR (1968). The fundamental nature of the genetic code: prebiotic interactions between polynucleotides and polyamino acids or their derivatives. Proc Natl Acad Sci U S A.

[B95] Woese CR, Dugre DH, Dugre SA, Kondo M, Saxinger WC (1966). On the fundamental nature and evolution of the genetic code. Cold Spring Harb Symp Quant Biol.

[B96] Woese CR, Dugre DH, Saxinger WC, Dugre SA (1966). The molecular basis for the genetic code. Proc Natl Acad Sci U S A.

[B97] Saxinger C, Ponnamperuma C, Woese C (1971). Evidence for the interaction of nucleotides with immobilized amino-acids and its significance for the origin of the genetic code. Nat New Biol.

[B98] Reuben J, Polk FE (1980). Nucleotide-amino acid interactions and their relation to the genetic code. J Mol Evol.

[B99] Khaled MA, Mullins DW, Lacey JC (1984). Binding constants of phenylalanine for the four mononucleotides. J Mol Evol.

[B100] Lacey JC, Mullins DW, Khaled MA (1984). The case for the anticode. Orig Life.

[B101] Mullins DW, Lacey JC (1980). Genetic code correlations: differential rates of non-enzymatic activation of hydrophobic amino acids by ATP. Biochem Biophys Res Commun.

[B102] Reuben J (1978). Chemically-selective nucleotide-amino acid interactions in aqueous solution. A PMR study. FEBS Lett.

[B103] Patel DJ, Suri AK (2000). Structure, recognition and discrimination in RNA aptamer complexes with cofactors, amino acids, drugs and aminoglycoside antibiotics. J Biotechnol.

[B104] Yarus M, Caporaso JG, Knight R (2005). Origins of the genetic code: the escaped triplet theory. Annu Rev Biochem.

[B105] Knight RD, Landweber LF (1998). Rhyme or reason: RNA-arginine interactions and the genetic code. Chem Biol.

[B106] Ellington AD, Khrapov M, Shaw CA (2000). The scene of a frozen accident. Rna.

[B107] Knight RD, Landweber LF (2000). Guilt by association: the arginine case revisited. Rna.

[B108] Majerfeld I, Yarus M (1998). Isoleucine:RNA sites with associated coding sequences. Rna.

[B109] Knight R, Yarus M (2003). Analyzing partially randomized nucleic acid pools: straight dope on doping. Nucleic Acids Res.

[B110] Majerfeld I, Puthenvedu D, Yarus M (2005). RNA affinity for molecular L-histidine; genetic code origins. J Mol Evol.

[B111] Majerfeld I, Yarus M (2005). A diminutive and specific RNA binding site for L-tryptophan. Nucleic Acids Res.

[B112] Lozupone C, Changayil S, Majerfeld I, Yarus M (2003). Selection of the simplest RNA that binds isoleucine. Rna.

[B113] Trifonov EN (2004). The triplet code from first principles. J Biomol Struct Dyn.

[B114] Copley SD, Smith E, Morowitz HJ (2005). A mechanism for the association of amino acids with their codons and the origin of the genetic code. Proc Natl Acad Sci U S A.

[B115] Szathmary E (1993). Coding coenzyme handles: a hypothesis for the origin of the genetic code. Proc Natl Acad Sci U S A.

[B116] Szathmary E (1999). The origin of the genetic code: amino acids as cofactors in an RNA world. Trends Genet.

[B117] Szathmary E, Maynard Smith J (1997). From replicators to reproducers: the first major transitions leading to life. J Theor Biol.

[B118] Di Giulio M (2004). The origin of the tRNA molecule: implications for the origin of protein synthesis. J Theor Biol.

[B119] Yarus M (1998). Amino acids as RNA ligands: a direct-RNA-template theory for the code's origin. J Mol Evol.

[B120] Altsein AD, Kverin NV (1980). On the origin of viral genetic systems. Zh Vsesoyuz Chim Ob im Mendeleeva.

[B121] Altstein (1987). Origin of the genetic system: the progene hypothesis. Mol Biol (Moscow).

[B122] Altstein AD (1992). The protocellular concept of the origin of viruses. Semin Virol.

[B123] Poole AM, Jeffares DC, Penny D (1998). The path from the RNA world. J Mol Evol.

[B124] Fredrick K, Noller HF (2003). Catalysis of ribosomal translocation by sparsomycin. Science.

[B125] Szathmary E, Demeter L (1987). Group selection of early replicators and the origin of life. J Theor Biol.

[B126] Zintzaras E, Santos M, Szathmary E (2002). "Living" under the challenge of information decay: the stochastic corrector model vs. hypercycles. J Theor Biol.

[B127] Sun L, Cui Z, Gottlieb RL, Zhang B (2002). A selected ribozyme catalyzing diverse dipeptide synthesis. Chem Biol.

[B128] Zhang B, Cech TR (1997). Peptide bond formation by in vitro selected ribozymes. Nature.

[B129] Zhang B, Cech TR (1998). Peptidyl-transferase ribozymes: trans reactions, structural characterization and ribosomal RNA-like features. Chem Biol.

[B130] Robertson MP, Knudsen SM, Ellington AD (2004). In vitro selection of ribozymes dependent on peptides for activity. Rna.

[B131] Cui Z, Sun L, Zhang B (2004). A peptidyl transferase ribozyme capable of combinatorial peptide synthesis. Bioorg Med Chem.

[B132] Kumar RK, Yarus M (2001). RNA-catalyzed amino acid activation. Biochemistry.

[B133] Delarue M, Poch O, Tordo N, Moras D, Argos P (1990). An attempt to unify the structure of polymerases. Protein Eng.

[B134] Joyce CM, Steitz TA (1994). Function and structure relationships in DNA polymerases. Annu Rev Biochem.

[B135] Aravind L, Leipe DD, Koonin EV (1998). Toprim--a conserved catalytic domain in type IA and II topoisomerases, DnaG-type primases, OLD family nucleases and RecR proteins. Nucleic Acids Res.

[B136] Force A, Cresko WA, Pickett FB, Proulx SR, Amemiya C, Lynch M (2005). The origin of subfunctions and modular gene regulation. Genetics.

[B137] Lynch M, Force A (2000). The probability of duplicate gene preservation by subfunctionalization. Genetics.

[B138] Darnell J, Lodish H, Baltimore D (1990). Molecular Cell Biology.

[B139] Wohlgemuth I, Beringer M, Rodnina MV (2006). Rapid peptide bond formation on isolated 50S ribosomal subunits. EMBO Rep.

[B140] Leipe DD, Aravind L, Koonin EV (1999). Did DNA replication evolve twice independently?. Nucleic Acids Res.

[B141] Forterre P (2002). The origin of DNA genomes and DNA replication proteins. Curr Opin Microbiol.

[B142] Forterre P (2005). The two ages of the RNA world, and the transition to the DNA world: a story of viruses and cells. Biochimie.

[B143] Iyer LM, Koonin EV, Aravind L (2003). Evolutionary connection between the catalytic subunits of DNA-dependent RNA polymerases and eukaryotic RNA-dependent RNA polymerases and the origin of RNA polymerases. BMC Struct Biol.

[B144] Forterre P (2006). Three RNA cells for ribosomal lineages and three DNA viruses to replicate their genomes: a hypothesis for the origin of cellular domain. Proc Natl Acad Sci U S A.

[B145] Noller HF (2004). The driving force for molecular evolution of translation. Rna.

[B146] Illangasekare M, Kovalchuke O, Yarus M (1997). Essential structures of a self-aminoacylating RNA. J Mol Biol.

[B147] Illangasekare M, Sanchez G, Nickles T, Yarus M (1995). Aminoacyl-RNA synthesis catalyzed by an RNA. Science.

[B148] Murakami H, Saito H, Suga H (2003). A versatile tRNA aminoacylation catalyst based on RNA. Chem Biol.

[B149] Lohse PA, Szostak JW (1996). Ribozyme-catalysed amino-acid transfer reactions. Nature.

[B150] Illangasekare M, Yarus M (1999). A tiny RNA that catalyzes both aminoacyl-RNA and peptidyl-RNA synthesis. Rna.

[B151] Nissen P, Hansen J, Ban N, Moore PB, Steitz TA (2000). The structural basis of ribosome activity in peptide bond synthesis. Science.

[B152] Polacek N, Gaynor M, Yassin A, Mankin AS (2001). Ribosomal peptidyl transferase can withstand mutations at the putative catalytic nucleotide. Nature.

[B153] Thompson J, Kim DF, O'Connor M, Lieberman KR, Bayfield MA, Gregory ST, Green R, Noller HF, Dahlberg AE (2001). Analysis of mutations at residues A2451 and G2447 of 23S rRNA in the peptidyltransferase active site of the 50S ribosomal subunit. Proc Natl Acad Sci U S A.

[B154] Rodnina MV, Beringer M, Wintermeyer W (2007). How ribosomes make peptide bonds. Trends Biochem Sci.

